# A BioID-based approach uncovers the interactome of hexose-6-phosphate dehydrogenase in breast cancer cells and identifies anterior gradient protein 2 as an interacting partner

**DOI:** 10.1186/s13578-025-01388-9

**Published:** 2025-04-25

**Authors:** Gabriele Sakalauskaite, Michael Weingartner, Sophie Ebert, Gina Boot, Thomas Bock, Julia Birk, Maria Tsachaki, John W. Gallon, Salvatore Piscuoglio, Alex Odermatt

**Affiliations:** 1 Division of Molecular and Systems Toxicology, Department of Pharmaceutical Sciences, University of Basel, Klingelbergstrasse 50, 4056 Basel, Switzerland; 2 Visceral Surgery and Precision Medicine Research Laboratory, Department of Biomedicine, University of Basel, Basel, Switzerland; 3 Proteomics Core Facility, Biozentrum, University of Basel, 4056 Basel, Switzerland; 4 IRCCS Humanitas Research Hospital, Rozzano, 20089 Milan, Italy

**Keywords:** Anterior gradient protein 2, Hexose-6-phosphate dehydrogenase, Endoplasmic reticulum, Protein–protein interaction, Proximity biotinylation, Breast cancer

## Abstract

**Background:**

Hexose-6-phosphate dehydrogenase (H6PD) catalyzes the first two steps of the pentose-phosphate-pathway (PPP) within the endoplasmic reticulum, generating NADPH. H6PD modulates essential physiological processes, including energy and redox metabolism. Its sole reported interacting partner is 11β-hydroxysteroid dehydrogenase 1 (11β-HSD1), utilizing NADPH to reactivate glucocorticoids, linking energy status with hormonal response. Previous studies showed that loss of H6PD affects breast cancer cell properties, independent of 11β-HSD1. It remains unknown whether this is due to impaired concentrations of NADPH or PPP products downstream of H6PD. To gain insight into novel roles and pathways influenced by this enzyme, we aimed to assess the H6PD interactome.

**Results:**

We adapted the proximity-dependent Biotin Identification (BioID) method to identify novel H6PD interacting partners. First, we validated the method and confirmed the known interaction between H6PD and 11β-HSD1. Next, we constructed a triple-negative breast cancer MDA-MB-231 cell clone stably expressing a H6PD-biotin ligase fusion protein. Enriched biotinylated proteins were analyzed by mass-spectrometry and potential candidates assessed further by co-immunoprecipitation and functional assays. The resulting interactome revealed proteins of the calreticulin/calnexin cycle, unfolded-protein response (UPR) and chaperone activation pathways. Due to its known association with breast cancer, we examined the PDI Anterior gradient protein 2 (AGR2) as H6PD interacting partner. Gene set enrichment analysis revealed multiple overlapping pathways enriched in breast cancer tissues with relatively high H6PD and AGR2 expression. These included glycolysis, fatty acid metabolism, hypoxia, angiogenesis and epithelial to mesenchymal transition. Co-immunoprecipitation (Co-IP) from MCF7 cells confirmed a physical interaction between H6PD and AGR2. ARG2 knockdown in these cells increased H6PD protein levels but decreased activity. Coexpression with AGR2 in HEK-293 cells did not affect expression but enhanced H6PD activity.

**Conclusion:**

BioID was successfully applied in the endoplasmic reticulum to identify AGR2 as H6PD interactor. This was confirmed using Co-IP from MCF7 cells endogenously expressing both proteins. The results indicate that AGR2 controls H6PD protein expression and enhances its activity. Whether higher H6PD activity due to increased AGR2 expression promotes a more aggressive cancer cell phenotype, for example by altering energy metabolism, Ca^2+^-related processes or UPR and chaperone activation pathways, warrants further investigations.

**Supplementary Information:**

The online version contains supplementary material available at 10.1186/s13578-025-01388-9.

## Introduction

Breast cancer is the most common malignant tumor diagnosed in women, with the highest mortality rate worldwide [1]. Endoplasmic reticulum-stress, activation of the unfolded protein response (UPR), and alterations in Ca^2+^-homeostasis have been associated with breast cancer progression [2–5]. Furthermore, tumor cells show several metabolic adaptations such as favoring an acidic microenvironment, anaerobic and aerobic glycolysis, and exhibiting an enhanced activity of the pentose-phosphate pathway (PPP) to fulfill their metabolic requirements. A high PPP activity is needed in rapidly proliferating cells for the production of ribose-5-phosphate (R5P) used for the synthesis of nucleotides and nucleic acids that are incorporated into DNA as well as for generating NADPH for the synthesis of fatty acids and cholesterol, and for detoxification reactions [6].

Most of the information currently available on the role of the PPP in cancer cells, and on PPP in general, comes from work on the cytosolic pathway. However, an alternative PPP exists also in the endoplasmic reticulum, with hexose-6-phosphate dehydrogenase (H6PD) catalyzing the first two steps, *i.e.* the conversion of glucose-6-phosphate (G6P) to 6-phosphogluconolactone (6PGL) by its dehydrogenase domain (thereby reducing NADP^+^ to NADPH), and further to 6-phosphogluconate (6PG) by its lactonase domain [7–9]. In contrast to the cytosolic glucose-6-phosphate dehydrogenase (G6PD), the luminal homolog H6PD is more promiscuous and accepts, besides G6P, glucose-6-sulfate (G6S), galactose-6-phosphate (Gal6P) and glucosamine-6-phosphate [7, 10]. The luminal PPP remains poorly understood despite increasing evidence for its role in modulating different properties in various cell types. For example, H6PD was shown to impact the regulation of endoplasmic reticulum-stress, Ca^2+^-homeostasis and luminal redox balance in breast cancer cell lines [11] as well as in skeletal muscles [12]. With respect to breast cancer cells, it was demonstrated that knockdown of H6PD decreased proliferation and migration of triple-negative (progesterone receptor (PR), estrogen receptor-α (ER), HER2 receptor (HER2)) SUM159 cells, in PR^+^/ER^+^/HER2^−^ MCF7 cells, and in triple-negative MDA-MB-231 cells [11].

However, the enzymes and reactions in the endoplasmic reticulum lumen of breast cancer cells that depend on NADPH or on the products of the luminal PPP remain unknown. Because NADP^+^ and NADPH cannot freely permeate and are not transported across the endoplasmic reticulum membrane, their luminal concentrations need to be regulated independent of the cytosolic pyridine-nucleotide pool [13–15]. Regarding the luminal PPP products, it remains unknown how their pools are regulated, warranting further research. So far, the interaction of H6PD with 11β-hydroxysteroid dehydrogenase type 1 (11β-HSD1), converting physiologically inactive cortisone to the potent glucocorticoid cortisol (or 11-dehydrocorticosterone to corticosterone in rodents), thereby providing a functional coupling between cellular G6P levels and glucocorticoid-dependent hormonal response, represents the most extensively investigated NADPH-dependent process within the endoplasmic reticulum [16–22]. However, most breast cancer cell lines, including MDA-MB-231 and MCF7, do not express 11β-HSD1 and no other proteins directly interacting with H6PD have been described so far.

Besides specific cofactors and post-translational modifications, interacting partners essentially modulate a protein’s functions [23–28]. Their identification is indispensable to understand protein functions [29], and increasing efforts to understand protein–protein interactions revealed the protein interactome as a new class of potential drug targets [30, 31]. Given the restricted knowledge on 11β-HSD1-independent H6PD functions, we aimed to assess the H6PD interactome in breast cancer cells that are devoid of 11β-HSD1 expression. For this purpose, we applied the proximity-dependent Biotin Identification (BioID) method to screen for neighboring proteins of H6PD within the endoplasmic reticulum. The underlying principle of BioID includes a mutated (R118G), promiscuous biotin ligase (BirA*) that is fused to a bait protein (here H6PD) [32–34]. Biotinylation of proteins in the proximity of the bait protein is achieved by expression of the fusion-protein in the presence of biotin in the cell culture medium. Neighboring proteins bearing biotinylated lysine residues can then be detected by immunochemical or mass-spectrometry (MS) methods.

The identified potential interactors of H6PD were then subjected to pathway analysis to gain insight into cellular processes that might be affected by this enzyme. Due to its high score in the BioID screening and its reported role in breast cancer, anterior gradient protein 2 (AGR2) was selected for a more detailed analysis. Gene enrichment analysis was performed to investigate the pathway enrichment in breast cancer tissue in relation to H6PD and AGR2 protein expression. To confirm AGR2 as interacting partner of H6PD, we performed co-immunoprecipitation (Co-IP) experiments. Finally, to begin to understand the functional consequences of the identified interaction of H6PD and AGR2, we performed gene silencing and overexpression experiments and determined H6PD expression and activity.

## Materials and methods

### Chemicals

All chemicals were purchased from Merck (former Sigma-Aldrich, Darmstadt, Germany) unless stated otherwise.

### Cell lines

MDA-MB-231, MCF7, HEK-293 and SUM-159 cell lines were purchased from American Type Culture Collection (ATCC, Manassas, VA, USA), tested regularly for the absence of mycoplasma contamination, and cultured under standard conditions (37 °C, 5% CO_2_). MDA-MB-231 cells were cultured in Roswell Park Memorial Institute (RPMI)−1640 medium supplemented with 10% fetal bovine serum (FBS), MCF7 cells in Dulbecco’s Modified Eagle’s Medium (DMEM) containing 2 mM L-glutamine, 4.5 g L^−1^ glucose, 10% FBS and non-essential amino acid mixture, and SUM-159 cells in Ham’s F-12 Nutrient Mix (Thermo Fisher Scientific, Waltham, MA, USA) containing 5% FBS and 5 µg mL^−1^ bovine pancreas insulin (#I6634). HEK-293 cells were cultured in DMEM containing 2 mM L-glutamine, 4.5 g L^−1^ glucose, non-essential amino acid mixture and 10% FBS. All cell culture media were supplemented with 10 mM hydroxyethylpiperazinethanesulfonic acid (HEPES), pH 7.4, 100 U mL^−1^ penicillin and 0.1 mg mL^−1^ streptomycin.

### Expression constructs and transfection

The BioID-fusion protein construct H6PD-BirA*-HA (Additional Table 1) was generated by PCR amplification, inserting full-length H6PD coding sequence from a donor vector into a pcDNA3.1 MCS-BirA(R118G)-HA plasmid (#36047, Addgene, Watertown, MA, USA). The C-terminally FLAG-tagged 11β-HSD1 (11β-HSD1-FLAG) construct in pcDNA3.1 was described earlier [35]. MDA-MB-231 cells were transfected using Lipofectamine 2000 reagent (#11668030, Thermo Fisher Scientific). HEK-293 cells were transiently transfected with H6PD-MYC plasmid [19] with and without AGR2-FLAG (#OHu09079;NM006408, in pcDNA3.1; Genscript, Piscataway, NJ, USA) using Ca^2+^-phosphate precipitation as described earlier [36]. Empty pcDNA3.1 vector served as control. Cells were harvested 24 h post-transfection for isolation of the microsomal fraction.

Gene silencing experiments were performed using Lipofectamine RNAiMax (#13778075, Thermo Fisher Scientific) and the following target siRNA (Microsynth, Balgach, St. Gallen, Switzerland) sequences: *H6PD* 5′-GGG CUA CGC UCG GAU CUU G-3′; *AGR2*: 5'-GAA GCU CUA UAU AAA UCC A-3'; Control 5′-UGG UUU ACA UGU UUU CUG A-3′. For experiments using whole cell lysates, 350′000 MDA-MB-231 or MCF7 cells were seeded in 6-well plates and transfected using 29 nM siRNA in combination with 2.9 µL Lipofectamine RNAiMAX reagent. Control siRNA served as control. For experiments using microsomes of MCF7 cells, reverse transfection was performed at a cell density of 1.5 × 10^6^, using 40 µM siRNA and 36 µL of RNAiMax reagent.

### Generation of cell lines stably expressing H6PD-BirA*-HA and/or 11β-HSD1-FLAG and biotinylation

MDA-MB-231 cells transfected with plasmid encoding H6PD-BirA*-HA or 11β-HSD1-FLAG were were selected with media containing 1000 µg mL^−1^ geneticin (G418, #13200, Cayman Chemical) and 0.5 µg mL^−1^ puromycin, respectively. Clones were screened for correct localization and expression of H6PD-BirA*-HA and 11β-HSD1-FLAG by immunoblotting and immunohistochemical analysis. Cells stably expressing H6PD-BirA*-HA were cultured in presence of 50 µM biotin (#B4639, Sigma Aldrich) for 72 h, followed by cell lysis.

### Sample preparation for mass-spectrometry analysis

MDA-MB-231 or stably expressing H6PD-BirA*-HA cells were cultured in the absence (−) or presence (+) of 50 µM biotin for 72 h. Cells were lysed using Radioimmunoprecipitation assay (RIPA) buffer (#89900, Thermo Fisher Scientific) containing protease inhibitor cocktail (#11836153001, Merck) and incubated in a thermo-shaker for 10 min at 4 °C at 1000 rpm, followed by centrifugation for 10 min at 16′000 × g. The supernatant was collected and protein concentration determined by the bicinchoninic acid assay (#23225, Thermo Fisher Scientific). To enrich biotinylated proteins, 60 µL streptavidin magnetic beads (#88817, Thermo Fisher Scientific) were mixed thoroughly and washed 3 times with 1 mL washing buffer (20 mM KH_2_PO_4_, 0.15 M NaCl). The beads were then incubated for 2 h at 4 °C under continuous rotation with standardized amount of total protein (1–1.5 mg) and washed 4 times with 1 mL RIPA buffer. Finally, the beads were washed 5 times using HNN buffer (50 mM HEPES pH 7.5, 150 mM NaCl, 5 mM EDTA, 50 mM NaF).

For on-bead digestion, 142.5 µL 100 mM ammonium bicarbonate (ABC) were added to the beads, followed by mixing and sonicating. Subsequently, 7.5 µL of 200 mM tris(2-carboxyethyl)phosphine (TCEP) were added, samples mixed, 3.2 µL of 750 mM chloracetamide (CAA) added and samples mixed again. The beads were incubated in the dark for 1 h at 37 °C on a thermo-shaker at 600 rpm, followed by adding 1 µg trypsin (# V5111, Promega, Fitchburg, WI, USA), gently mixing and incubated overnight (o/n) at 37 °C with continuous shaking at 800 rpm. Beads were then subjected to magnetic separation and supernatant was collected and stored at 4 °C. The beads were incubated for 2 h in 150 µL ABC buffer supplied with 1 µg trypsin. The beads were separated, and the aqueous phase was collected and pooled with the first aspirated fraction and stored at 4 °C. Beads were then mixed with 200 µL 0.1% trifluoroacetic acid. After shaking for 5 min at 1000 rpm, followed by bead separation, the acidic phase was pooled with the previous fractions. Protein purification and desalting of the pooled fractions were achieved through the application of C-18 MiniSpin® columns (#SEM SS18 V, The Nest Group, Southborough, MA, USA). After purification and desalting, the peptides were dried by centrifugal evaporation for 2 h using a CentriVap concentrator (Kansas City, MO, USA). Dried peptides were stored at − 20 °C until analysis.

### MS data acquisition

Dried peptides were dissolved in 0.1% aqueous formic acid (0.25 mg mL^−1^) prior to subjecting 0.25 μg of peptides to LC–MS/MS analysis using a dual pressure LTQ-Orbitrap Elite mass-spectrometer (MS) connected to an electrospray ion source (Thermo Fisher Scientific) and a custom-made column heater set to 60 °C. Peptide separation was carried out using an EASY nLC-1000 system (Thermo Fisher Scientific) equipped with a RP-HPLC column (75 μm × 30 cm) packed in house with C18 resin (ReproSil-Pur C18–AQ, 1.9 μm resin; Dr. Maisch GmbH, Germany). Peptides were separated using a step wise linear gradient from 95% solvent A (0.1% formic acid, in water) and 5% solvent B (80% acetonitrile, 0.1% formic acid, in water) to 35% solvent B over 50 min, to 50% solvent B over 10 min, to 95% solvent B over 2 min, and to 95% solvent B over 18 min at a flow rate of 0.2 µL/min^−1^. The data acquisition mode was set to obtain one high resolution MS scan at a resolution of 240′000 full width at half maximum (at 400 m/*z*, MS1) followed by MS/MS (MS2) scans in the linear ion trap of the 20 most intense MS signals. The charged state screening modus was enabled to exclude unassigned and singly charged ions and the dynamic exclusion duration was set to 30 s. The collision energy was set to 35%, and one microscan was acquired for each spectrum.

### Protein identification and label-free quantification

The acquired raw-files were imported into the Progenesis QI software (v2.0, Nonlinear Dynamics Limited, Newcastle upon Tyne, UK), which was used to extract peptide precursor ion intensities across all samples applying the default parameters. The generated mgf-files were searched using MASCOT (Matrix Science, Boston, MA, USA) against a decoy database containing normal and reverse sequences of the concatenated Homo sapiens (UniProt, 26 th April 2016) proteome including commonly observed contaminants (in total 141′240 sequences) generated using the SequenceReverser tool from the MaxQuant software (Version 1.0.13.13, Max Planck Institute of Biochemistry, Martinsried, Germany). The following search criteria were used: full tryptic specificity was required (cleavage after lysine or arginine residues, unless followed by proline); 3 missed cleavages were allowed; carbamidomethylation (C) was set as fixed modification; oxidation (M) and protein N-terminal acetylation were applied as variable modifications; mass tolerance of 10 ppm (precursor) and 0.6 Da (fragments) was set. The database search results were filtered using the ion score to set the false discovery rate (FDR) to 1% on the peptide and protein level, respectively, based on the number of reverse protein sequence hits in the datasets. Quantitative analysis results from label-free quantification were normalized and statistically analyzed using the SafeQuant R package v.2.3.4 (https://github.com/eahrne/SafeQuant/; [37] to obtain protein relative abundances. This analysis included summation of peak areas per protein and LC–MS/MS run followed by calculation of protein abundance ratios. Only isoform specific peptide ion signals were considered for quantification. The summarized protein expression values were used for statistical testing of differentially abundant proteins between conditions. Here, empirical Bayes moderated t-tests were applied, as implemented in the R/Bioconductor limma package (http://bioconductor.org/packages/release/bioc/html/limma.html). The resulting p-values were adjusted for multiple testing using the Benjamini–Hochberg method.

All LC–MS/MS analysis runs were acquired from samples of three independent experiments. To meet additional assumptions (normality and homoscedasticity) underlying the use of linear regression models and Student’s t-test, MS-intensity signals were transformed from the linear to the log-scale. Unless stated otherwise, linear regression was performed using the ordinary least square (OLS) method as implemented in base package of R v.3.1.2 (http://www.R-project.org/).

The sample size of three biological replicates was chosen assuming a within-group MS signal coefficient of variation of 10%. When applying a two-sample, two-sided Student’s t-test this was found to give adequate power (80%) to detect protein abundance fold changes higher than 1.65, per statistical test. The statistical package used to assess protein abundance changes, SafeQuant, employs a moderated t-test, which has been shown to provide higher power than the Student’s t-test. No simulations were conducted to assess power, upon correction for multiple testing (Benjamini–Hochberg correction), as a function of different effect sizes and assumed proportions of differentially abundant proteins.

### Protein expression—immunoblotting

Underlying procedures for cell lysis, protein extraction, sodium dodecyl sulfate polyacrylamide gel electrophoresis (SDS-PAGE), immunoblotting blotting and detection for all proteins examined in this study, except biotinylated proteins, were previously described [38]. To detect biotinylated proteins, polyvinylidene fluoride (PVDF) membranes were blocked in bovine serum albumin (BSA, 5%) in Tris-buffered saline with Tween 20 (BSA-TBST) for 1 h and incubated o/n at 4 °C with streptavidin–horseradish peroxidase (HRP) at a dilution 1:10,000. Then, membranes were washed 3 times for 15 min in TBS-T, followed by incubation in adult bovine serum blocking buffer (ABS; 10% adult bovine serum, 1% Triton X-100 in phosphate buffered saline; PBS) for 5 min. Membranes were then washed 3 times for 1 min in PBS and analyzed using enhanced chemiluminescence substrate (#WBKLS0500, Merck). Primary and secondary antibodies to detect the proteins of interest are listed in Additional Table 2. Primary antibodies were used at dilutions 1:500–1:2′000, secondary antibodies at 1:2′000–1:4′000. Proteins (10–35 µg) were separated by SDS-PAGE. Densitometry analysis, where appropriate, was carried out using ImageJ software (version 1.53n, RRID:SCR_003070).

### TCGA data analysis

TCGA breast invasive carcinoma (BRCA) gene expression data (log2(RSEM-normalized counts + 1)) by RNAseq (polyA + IlluminaHiSeq) for the genes *AGR2* and *H6PD* were downloaded via Xenabrowser in February 2024 (https://xenabrowser.net/). TCGA samples with non-missing *AGR2* and *H6PD* expression values were reduced to 1097 primary tumor samples and 114 solid normal tissues within the BRCA cohort.

Annotation of PR, ER and HER2 Status was derived from ‘Supplementary Tables 1–4.xls’ of The Cancer Genome Atlas Network [39]. ER^−^/PR^−^/HER2^−^ were labelled as a ‘triple-negative’ subtype (n = 123) and ER^−^/PR^−^/HER2^+^ as ‘HER2-enriched’ (n = 30). Overall survival information for this cohort with Fragments Per Kilobase of transcript per Million mapped reads (FPKM) for each gene were retrieved from protein atlas (Version: 23.0 https://www.proteinatlas.org/ENSG00000106541-AGR2/pathology/breast+cancer; https://www.proteinatlas.org/ENSG00000049239-H6PD/pathology/breast+cancer). The number of patients with non-missing values included in survival analysis are indicated in the figures. Comparisons of numerical variables (e.g. individual gene expression) were performed using the Mann–Whitney U test. Correction for multiple testing was performed using the Benjamini–Hochberg method. To assess the correlation of gene expression, Spearman’s correlation was calculated using all of the samples or with the samples divided according to the indicated groups. Stratification of *AGR2* and *H6PD* expression in to high and low groups of expression for overall survival analysis was performed using optimal cut-off values as calculated by the “surv-cutpoint” function of the survminer package, based on the maximally selected rank statistics algorithm [40, 41]. Kaplan–Meier survival curves were used to investigate survival times, and differences between high and low groups were analyzed using a log-rank test. All statistical tests were two-sided unless otherwise indicated, and p ≤ 0.05 was considered statistically significant.

### Gene set enrichment analysis

Proteomics data from tissues of 122 human breast cancer (BRCA) patients from the Clinical Proteomic Tumor Analysis Consortium (CPTAC) was downloaded from Linkedomics [42] (https://www.linkedomics.org/data_download/CPTAC-BRCA/ accessed 06.11.2024)) as log-transformed normalized ratios, alongside relevant clinical data including clinical assessment of hormone receptor status. Samples were split into ‘high’ and ‘low’ AGR2 or H6PD abundance groups by taking the top and bottom 25% of samples regarding abundance of the respective marker, to determine 31 “higher abundance” and 31 “lower abundance” samples. This method was chosen to determine relative high/low abundance due to the lack of available non-tumor tissue data in the CPTAC BRCA dataset. To assess differential abundance between the groups of interest, limma (v3.52.4) was used to fit a linear model and apply empirical Bayes moderation, both with and without adjustment for TNBC status, adjusting for multiple testing using the false discovery rate (FDR) method [43]. A logFC threshold of 1.5 and adjusted p value of 0.05 was used to determine differentially abundant proteins. Pathway enrichment analysis was performed on the moderated t-statistics determined from the limma analysis using fgsea (v1.24) [44]. Cancer Hallmark gene sets were downloaded from MSigDB [45] using MSigDBR (MSigDBR: MSigDB gene sets for multiple organisms in a tidy data format, R package version 7.5.1, https://CRAN.R-project.org/package=msigdbr).

### Reversible crosslinking and co-immunoprecipitation

Crosslinking followed by co-immunoprecipitation (Co-IP) was modified from previously reported studies [46, 47]. Briefly, MCF7 or MDA-MB-231 cells cultured in 10 cm dishes were washed twice with PBS. Intracellular crosslinking was performed by incubating cells with PBS containing 2 mM dithiobis(succinimidylpropionate) and 0.5 mM dithiobismaleimidoethane (DSP, #c1106; DTME, #c1138, ProteoChem, Hurricane, UT, USA). After incubating for 45 min at room temperature, cells were washed twice with PBS, followed by quenching of the crosslinking-reaction using PBS containing 20 mM Tris–HCl, pH 7.5, and 5 mM L-cysteine for 15 min. Cells were washed twice with PBS and immediately lysed with Co-IP lysis buffer (50 mM Tris pH 7.5, 1% Triton X-100, 150 mM NaCl, 10% glycerol, 5 mM MgCl_2_ and protease inhibitor cocktail). The lysate was incubated for 10 min at 4 °C at 1000 rpm, and centrifuged at 16′000 × *g* for 10 min at 4 °C. Either 2 µg rabbit polyclonal anti-H6PD antibody (IP) or rabbit IgG Isotope control (IGG) were then added to 1 mg of supernatant protein. The protein-antibody mixture was incubated o/n at 4 °C under permanent shaking. Next, 25 µL of activated protein A magnetic beads (Cat#88846, Thermo Fisher Scientific) were added to the protein-antibody mixture and incubated for 1 h at room temperature while shaking. The beads were washed 5 times with 1 mL washing buffer (20 mM Tris, 0.5 M NaCl, 0.05% Tween-20) and once with 0.5 mL ultrapure water. 120 µL of SDS-PAGE sample buffer consisting of 70% v/v Co-IP lysis buffer, 5% v/v 0.5 M TCEP and 25% v/v SDS-PAGE loading sample buffer 4 × (240 mM Tris–HCl, pH 6.8, 40% glycerol, 277 mM SDS, 0.04% bromophenol blue) was added to the beads, followed by boiling for 10 min. The beads were separated and samples stored at − 20 °C until further use. Samples (30 µL) were subjected to SDS-PAGE to detect interacting proteins.

### Indirect immunofluorescence microscopy

Indirect immunofluorescence was performed as described previously [48]. The H6PD-BirA*-HA fusion protein was visualized using rat anti-HA antibody at a dilution of 1:150, followed by incubation with secondary goat anti-rat Alexa-555 antibody diluted 1:200 (Additional Table 2). Calnexin (CANX) was detected using rabbit polyclonal anti-CANX antibody, at 1:100 dilution, and secondary goat anti-rabbit Alexa-488 antibody at 1:200 dilution. Biotinylated proteins were detected using anti-streptavidin Alexa-488 antibody at 1:2000 dilution. Nuclei were stained using Hoechst 33342 (#62249, Thermo Fisher Scientific) diluted 1:2000. All images were acquired with the × 40 objective of the microscope (Leica DMI4000 B, Leica, Wetzlar, Germany).

### Preparation of microsomes and measurement of H6PD activity

Confluent 10 cm dishes of HEK-293 and MCF7 cells were washed with pre-warmed PBS, then with 1 mL ice cold PBS, followed by harvesting cells and centrifugation for 4 min at 150 × *g* at 4 °C. Washing was repeated, supernatant aspirated, and pelleted cells were resuspended in 600 µL homogenization buffer (20 mM Tris, pH 7.5, 50 mM KCl, 2 mM MgCl_2_, 0.25 M sucrose and protease inhibitor cocktail). Cells were transferred to a dounce homogenizer (2 mL, on ice) and processed by 20 strokes with periods of repeated cooling on ice every 5 strokes for 10 s. Debris were removed by centrifugation for 20 min at 12′000 × *g* at 4 °C, supernatant was transferred to a new tube and centrifuged for 1 h at 100′000 × *g* at 4 °C. Supernatant was aspirated and the microsomal pellet resuspended in 80 µL buffer (20 mM 3-(N-morpholino)propanesulfonic acid (MOPS), pH 7.2, 100 mM KCl, 20 mM NaCl, 1 mM MgCl_2_ and protease inhibitor cocktail). Microsomes were stored on ice before further use. Microsomes (20–30 µg of protein in 100 µL) were permeabilized by Triton X-100 (0.5% v/v) for 5 min and incubated with 0.4 mM NADP^+^ and 10 mM H6PD-specific substrate G6S (Glycoteam GmbH, Hamburg, Germany) [49]. H6PD activity was assessed by measuring the absorbance at 340 nm, representing NADPH formation, 1, 5, 10, 15, 20, 25 and 30 min after substrate and cofactor administration [50].

### Statistical analysis

If not stated otherwise, GraphPad Prism software 8.0 (GraphPad, La Jolla, CA, USA, RRID:SCR_002798) and Microsoft Excel (Microsoft, Redmond, WA, USA) were used for data analysis, with the respective statistical test as indicated. A *p*-value ≤ 0.05 was considered statistically significant.

## Results

### Generation of a MDA-MB-231 clone stably expressing H6PD-BirA*-HA and biotinylation of vicinal proteins

**Fig. 1:**
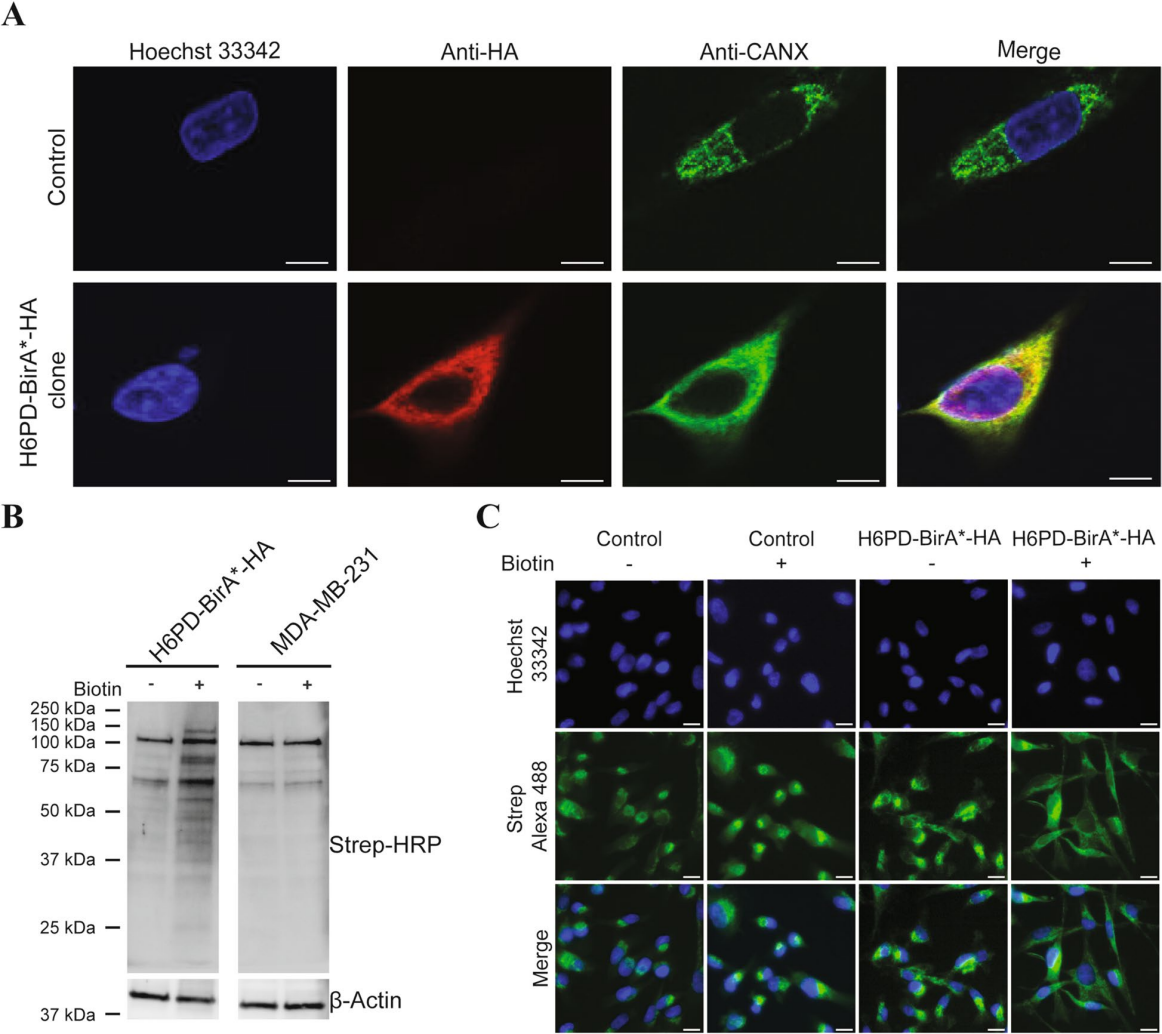
Characterization of the H6PD-BirA*-HA expressing MDA-MB-231 clone. The selected MDA-MB-231 cell clone expressing H6PD-BirA*-HA was analyzed by immunofluorescence and immunoblotting (**A**–**C**). **A** Confirmation of H6PD-BirA*-HA localization in the endoplasmic reticulum lumen using immunofluorescence in MDA-MB-231 cells stably expressing the H6PD-BirA*-HA fusion protein and in parental control cells. Staining was performed using rat anti-HA antibody, rabbit anti-CANX antibody, and the corresponding goat anti-rat Alexa-555 and goat anti-rabbit Alexa-488 secondary antibodies for visualization. In addition, nuclei were stained using Hoechst-33342. **B** Immunoblotting of lysates from parental MDA-MB-231 and H6PD-BirA*-HA expressing cells incubated for 72 h with or without 50 µM biotin. Membranes were probed with HRP labeled streptavidin (Strep-HRP) to detect biotinylated proteins. β-Actin served as loading control. **C** Cells were incubated with 50 µM biotin for 72 h where indicated. Biotinylation was qualitatively assessed using streptavidin Alexa-488 antibody. Representative images are shown, scale bars 15 µm

To identify novel interacting partners of H6PD within the endoplasmic reticulum, the previously developed BioID approach [32] was adapted by fusing the HA-tagged promiscuous biotin ligase BirA* to the C-terminus of H6PD (Additional Table 1). The TNBC cell line MDA-MB-231, endogenously expressing H6PD (Additional Fig. 1), was transfected with a plasmid encoding this fusion protein and subjected to geneticin selection. Individual, stably expressing clones were analyzed for the expression of the H6PD-BirA*-HA fusion protein and a sequence verified clone was selected for further experiments. First, the endoplasmic reticulum luminal localization of the H6PD-Bir*A-HA fusion protein was verified by indirect immunofluorescence analysis (Fig. 1A). Staining with anti-HA antibody and secondary Alexa-555 labeled antibody showed no unspecific signal in parental MDA-MB-231 control cells, and, importantly, the H6PD-Bir*A-HA fusion protein showed a typical reticular pattern and co-localized with the known endoplasmic reticulum luminal marker CANX. Next, biotinylation was achieved by adding biotin to the cell culture medium (final concentration: 50 µM), followed by incubation for 72 h. The selected MDA-MB-231 clone expressing luminal H6PD-BirA*-HA showed an increased proportion of biotinylated proteins compared to cells cultured without supplementation of biotin and compared to parental MDA-MB-231 cells, as visualized by immunoblotting and immunofluorescence (Fig. 1B, C). A typical endoplasmic reticulum pattern is only visible in H6PD-BirA*-HA cells that were exposed to biotin (Fig. 1C; see H6PD-BirA*-HA + biotin, strep Alexa-488 and merge). Together, this data indicated that H6PD-BirA*-HA is functional and localizes exclusively in the endoplasmic reticulum lumen.

### Testing the BioID approach in the endoplasmic reticulum and verification of the known H6PD-11β-HSD1 interaction

**Fig. 2:**
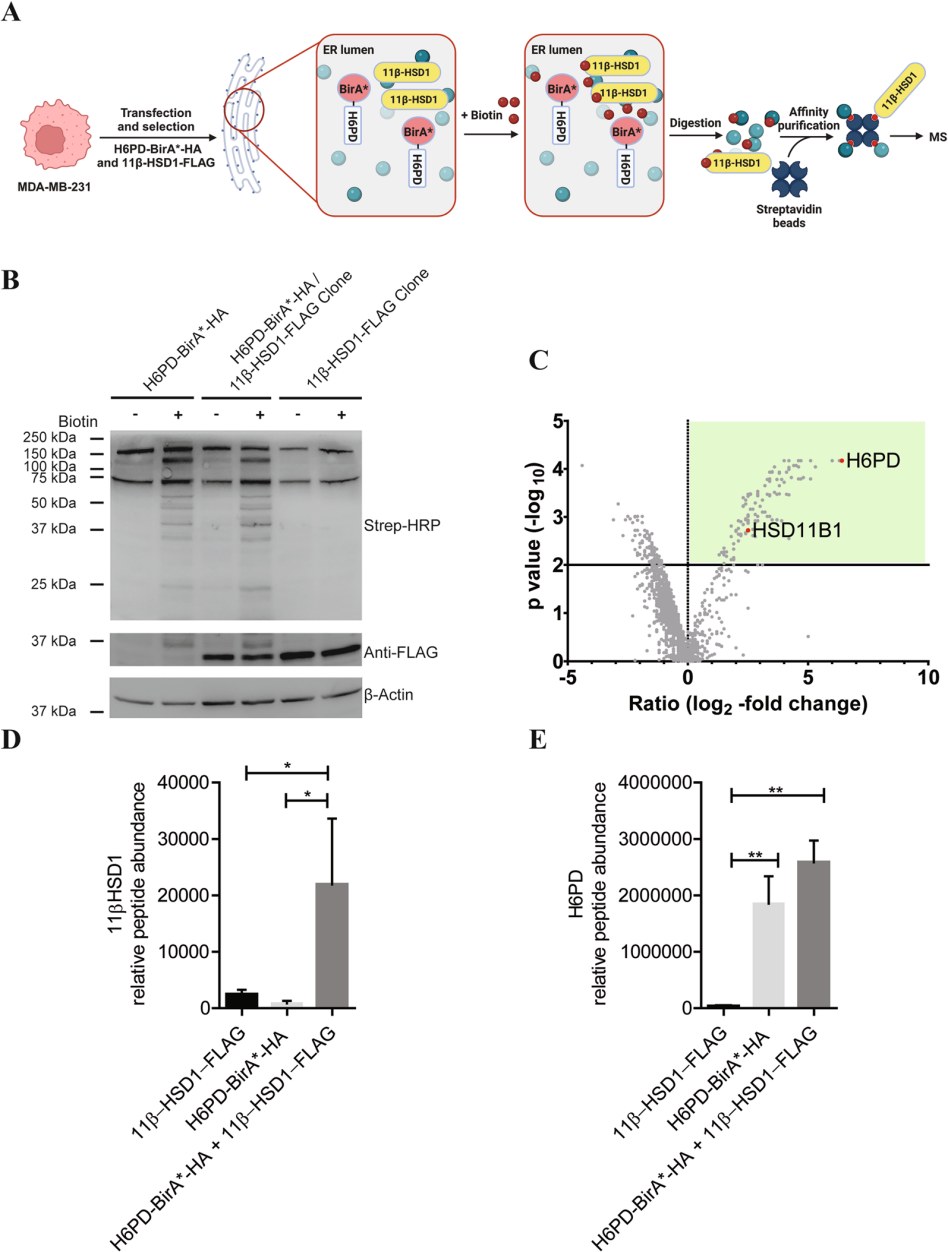
Identification of biotinylated 11β-HSD1 by MS from cells stably expressing H6PD-BirA*HA and 11β-HSD1-FLAG. **A** Scheme of the BioID approach for the detection of potential H6PD interactors. MDA-MB-231 cells were co-transfected with H6PD-BirA*-HA and 11β-HSD1-FLAG, followed by selection of a cell clone with stable expression of the two constructs. Cells stably expressing H6PD-BirA*-HA and 11β-HSD1-FLAG were incubated with 50 µM biotin for 72 h. Biotinylated proteins were then isolated from the cell lysate by affinity purification using streptavidin covalently coupled to magnetic beads and analyzed for the presence of biotinylated proteins by label-free quantitative MS. (Figure created using BioRender.com). **B** Immunoblotting of lysates from different clones expressing H6PD-BirA*-HA, H6PD-BirA*-HA/11β-HSD1-FLAG or 11β-HSD1-FLAG. Membranes were probed with HRP-labeled streptavidin to detect biotinylated proteins. β-Actin served as a loading control. **C** H6PD interactome proposed by the BioID approach using H6PD-BirA*-HA and 11β-HSD1-FLAG co-expressing MDA-MB-231 cells. Volcano plot representing the enrichment of peptides from H6PD and 11β-HSD1 in the biotinylated fraction of proteins from MDA-MB-231 cells co-expressing H6PD-BirA*-HA and 11β-HSD1-FLAG compared to parental MDA-MB-231 cells. Proteins were considered enriched if log_2_-fold change was ≥ 0 and the p-value was ≤ 0.01 (dashed line). Proteins that met this enrichment criteria, including 11β-HSD1, are highlighted by the green box and represent potential H6PD interactors. The x-axis of the presented volcano plot shows the ratio (log_2_-fold-change) of median protein abundance in samples from MDA-MB-231 cells stably co-expressing H6PD-BirA*-HA and 11β-HSD1-FLAG. The relative intensities of biotinylated peptides derived of **D** 11β-HSD1 and **E**) H6PD were determined in the cell clones as indicated. Data represent mean ± SD from three independent experiments. One-way ANOVA with Tukey’s post hoc test, p-values: * < 0.05, ** < 0.01

We next aimed to validate that the BioID approach successfully works within the endoplasmic reticulum by confirming the known interaction between H6PD and 11β-HSD1. For this purpose, we generated a cell clone stably co-expressing H6PD-BirA*-HA and 11β-HSD1-FLAG (Fig. 2A), because MDA-MB-231 cells do not endogenously express 11β-HSD1. The three cell clones expressing either H6PD-BirA*-HA, 11β-HSD1-FLAG, or co-expressing H6PD-BirA*-HA and 11β-HSD1-FLAG were then incubated in the absence or presence of biotin, subjected to affinity purification using streptavidin-conjugated magnetic beads and subsequently analyzed by immunoblotting (Fig. 2B). Whilst addition of biotin did not change the protein pattern for the 11β-HSD1-FLAG expression clone compared with its vehicle control, both the H6PD-BirA*-HA/11β-HSD1-FLAG and the H6PD-BirA*-HA clones showed substantially higher numbers of biotinylated proteins following biotin treatment with a similar pattern, demonstrating that biotinylation was due to H6PD-BirA*-HA activity (Fig. 2B). The MS analysis of the affinity-purified biotinylated proteins from MDA-MB-231 cells expressing H6PD-BirA*-HA and 11β-HSD1-FLAG resulted in the detection of 193 potential interactors, based on a p-value ≤ 0.01, including 11β-HSD1 (Fig. 2C). This shows the successful detection of a known H6PD interactor using the BioID method. In contrast, no enrichment of biotinylated 11β-HSD1 peptides could be observed in cells solely expressing 11β-HSD1-FLAG or in cells expressing H6PD-BirA*-HA only (Fig. 2D). Biotinylated H6PD peptides were enriched in the presence of H6PD-BirA*-HA but not in MDA-MB-231 cells expressing only 11β-HSD1-FLAG (Fig. 2E). These results indicate that the H6PD-BirA*-HA fusion protein biotinylated proteins in its close proximity and that this cell clone can be used to identify interactors of H6PD in the endoplasmic reticulum lumen.

### Proteomic analysis of biotinylated proteins potentially interacting with H6PD

**Fig. 3:**
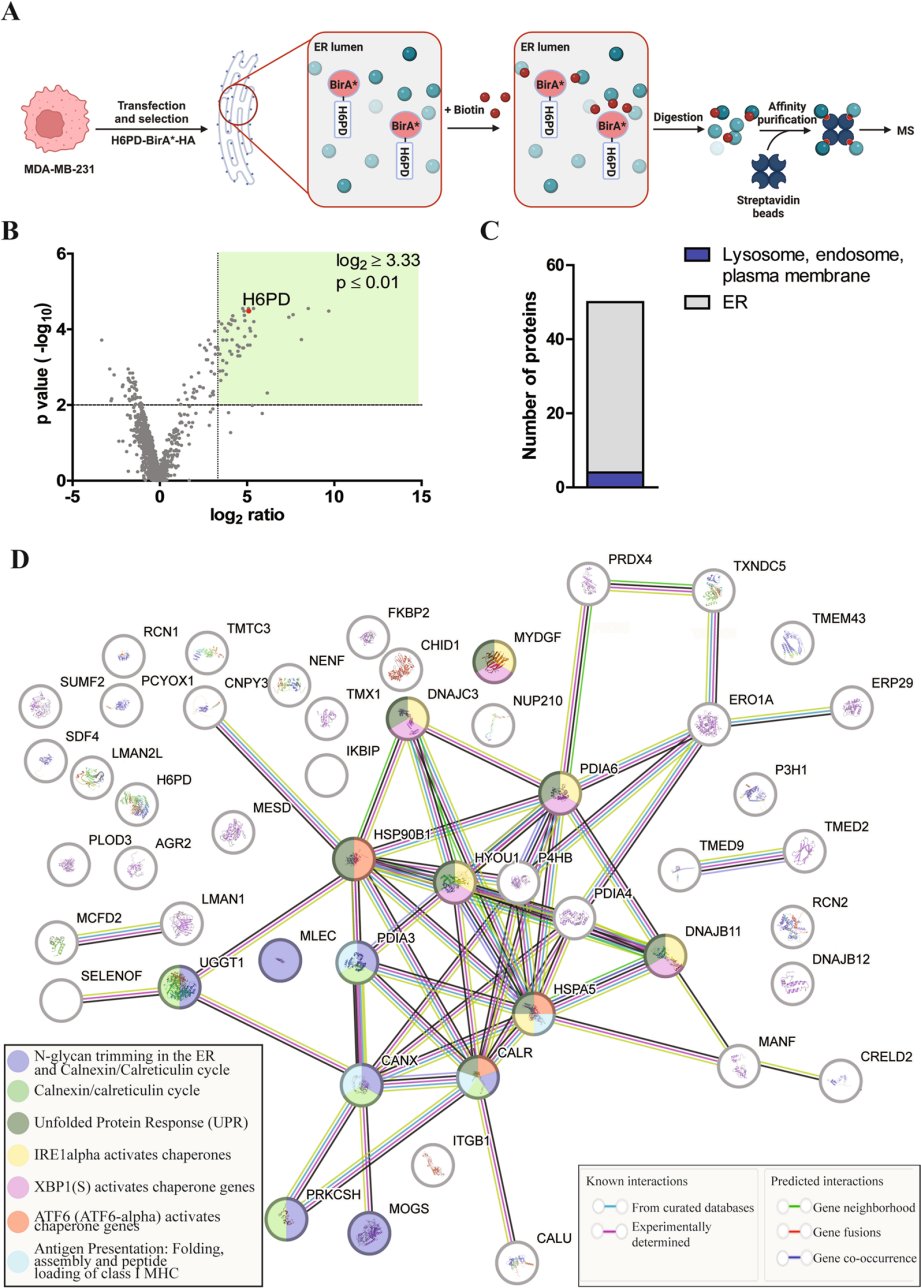
MS-based identification of proteins that were biotinylated by H6PD-BirA*-HA. **A** Schematic overview of the approach used to detect potential H6PD interactors in MDA-MB-231 cells expressing H6PD-BirA*-HA. Samples from cells stably expressing H6PD-BirA*-HA were incubated with 50 µM biotin for 72 h, biotinylated proteins were isolated from the lysate by affinity purification using streptavidin magnetic beads and analyzed for the presence of biotinylated proteins using label-free quantitative MS. (Figure created using BioRender.com). **B** H6PD interactome identified by the BioID approach in H6PD-BirA*-HA expressing MDA-MB-231 cells. In total, 50 proteins were enriched by a log_2_-fold change ≥ 3.33 and a p-value ≤ 0.01 in samples from the H6PD-BirA*-HA clone compared to control samples from MDA-MB-231 cells. Proteins that met these enrichment criteria are highlighted by the green box. The H6PD data point is depicted in red. **C** Subcellular localization of enriched proteins identified in B. The presented data were determined based on the results from three independent experiments. **D** Protein–protein interactions of the 50 enriched proteins predicted by the STRING database and pathway annotation based on the reactome pathway database. Predictions were made based on curated databases, experimentally determined information, gene neighborhood, co-occurrence or fusion as represented by lines between knots. Empty nodes represent proteins with no known or predicted 3D-structure, filled nodes indicate that 3D-structure is known or predicted. Color of the nodes represent the identified pathways to which the proteins belong to as described in the figure. Transparent nodes represent proteins that were not assigned to any of the listed pathways. Analysis was done with the highest confidence parameter (0.900)

Next, potential H6PD interactors were identified in MDA-MB-231 cells, expressing the H6PD-BirA*-HA fusion enzyme (Fig. 3A). Cells stably expressing H6PD-BirA*-HA and native MDA-MB-231 cells were cultured in the presence of biotin, lysed, and lysates subjected to affinity purification using streptavidin-conjugated magnetic beads. Bound biotinylated proteins were analyzed by label-free quantitative MS by comparing samples from H6PD-BirA*-HA expressing cells and parental MDA-MB-231 cells. In total, 1097 proteins could be assigned, including the bait protein H6PD (Fig. 3B, Additional Table 3), of which 1049 were identified by at least 2 different peptides and 904 by at least 3 different peptides. Among those, 82 proteins showed a log_2_-fold change ≥ 1 with a p-value ≤ 0.01. Furthermore, 50 of them were enriched in the samples from H6PD-BirA*-HA expressing cells by a factor of at least 10, corresponding to a log_2_-fold change ≥ 3.33, with a p-value ≤ 0.01 (Fig. 3B, Table 1).

With respect to the subcellular location, 46 out of the 50 most highly enriched proteins were allocated to the endoplasmic reticulum, and the remaining four were assigned to the lysosome, endosome and plasma membrane, thus passing the endoplasmic reticulum during their biosynthesis (Fig. 3C, Table 1). As shown in Table 1, the 50 most enriched proteins included nine PDI members, ten luminal (co-)chaperones, affiliated to different heat shock protein families and endoplasmic reticulum-resident lectin chaperones, seven Ca^2+^-binding proteins, and several proteins with different, not directly interrelated tasks of luminal glycoprotein quality control and processing.

**Table 1:**
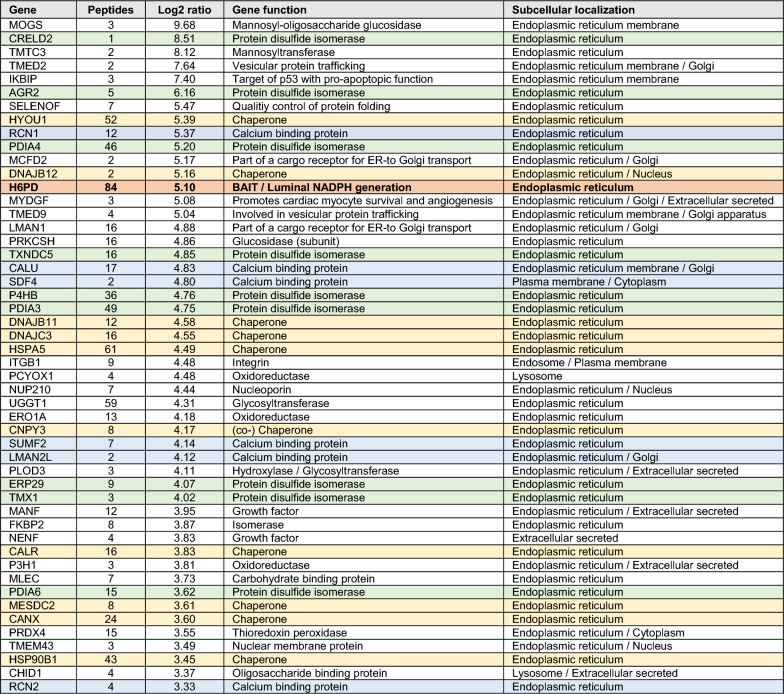
Top 50 biotinylated proteins identified by MS after IP using streptavidin-labeled magnetic beads

Samples from parental MDA-MB-231 and stably H6PD-BirA*-HA expressing cells were incubated with 50 µM biotin for 72 h and analyzed for the presence of biotinylated proteins by label-free quantitative MS. In H6PD-BirA*-HA samples, 50 proteins were enriched by a log_2_-fold change ≥ 3.33 and a p-value ≤ 0.01. PDI family members are highlighted in green, proteins with Ca^2+^-binding features are marked in blue, and endoplasmic reticulum-resident (co-)chaperones in yellow. The bait protein H6PD (bold) is marked in orange. The presented log_2_- fold change represents enrichment of identified peptides in the H6PD-BirA*-HA clone samples compared to MDA-MB-231 control samples. The numbers of peptides used for quantification of each protein is listed in the “Peptides” column. Assigned gene function and subcellular localization were adapted from UniProt (24 th December 2020) and sources cited in this work. The presented data were determined based on the results from three independent experiments

Next, the 50 most highly enriched targets were subjected to a putative interaction and pathway analysis using the STRING database, selecting the reactome pathway database (Fig. 3D, Additional Table 4). Many potential H6PD interactors detected by the BioID approach were predicted to interact with each other by the STRING database analysis and clustered in the same pathways. Interestingly, Mannosyl-oligosaccharide glucosidase (MOGS), Protein kinase C substrate 80 K-H (PRKCSH; also known as Glucosidase II Subunit Beta, GluIIBeta), CANX, PDI family A member 3 (PDIA3), Calreticulin (CALR), and UDP-glucose:glycoprotein glucosyltransferase (UGGT1) were predicted to form an interacting network clustering in the CANX/CALR cycle. Similarly, heat shock 70 kDa protein 5 (HSPA5), Hypoxia up-regulated protein 1 (HYOU1), DnaJ homolog subfamily C member 3 (DNAJC3), DnaJ homolog subfamily B member 11 (DNAJB11) and PDIA6 formed an interacting network and were assigned to the UPR, inositol requiring enzyme 1 alpha (IRE1α) and X-Box binding protein 1 (XBP1(S)) chaperone activation pathways. These results are particularly interesting because both UPR and Ca^2+^-related processes have been shown to contribute to breast cancer progression [51, 52], and H6PD was shown to affect UPR, Ca^2+^ homeostasis and redox balance [11].

For further investigations, we inspected the list of potential interactors by stringent selection parameters, *i.e.* log_2_-fold change and numbers of different peptides used for protein quantification. The PDI family member Anterior gradient protein 2 (AGR2) showed the highest log_2_-fold change (6.16) among the potential interacting candidates that were quantified by more than 3 peptides (Table 1, Additional Table 3). Because both AGR2 and H6PD have been associated with breast tumor promoting effects [11, 53], AGR2 was studied in more detail. First, the TCGA database was used to analyze *H6PD* and *AGR2* mRNA expression correlation in breast cancer subtypes, and contribution of the two genes for survival of breast cancer patients (Additional Fig. 2 and 3). H6PD mRNA was lower in primary, HER2-enriched, and triple-negative tumors, while *AGR2* mRNA was higher in HER2-enriched and ER-positive but lower in triple-negative tumors (Additional Fig. 2). Higher *H6PD* mRNA levels indicated higher survival in triple-negative cases, whereas higher AGR2 levels correlated with worse outcomes in both triple-negative and HER2-enriched cancers (Additional Fig. 3). However, the TCGA dataset analysis is limited by its reliance on mRNA expression, which often does not correlate with protein expression and/or enzyme activity. Therefore, TCGA analysis should be complemented by protein analysis and functional investigation where possible.

**Fig. 4:**
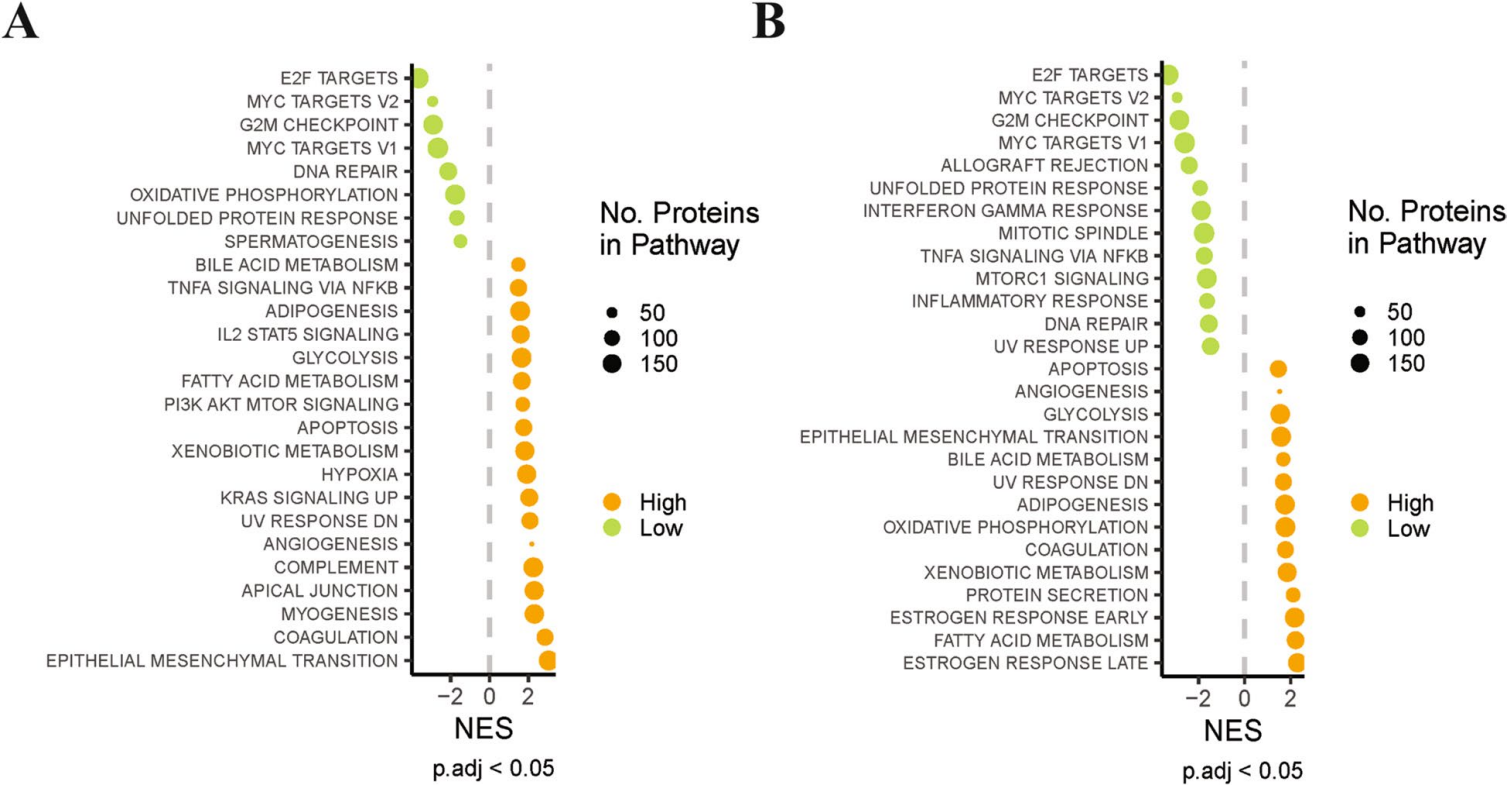
Pathway enrichment analysis indicating multiple overlapping pathways at high and low H6PD and AGR2 protein expression. **A** Gene set enrichment analysis showing normalized enrichment score (NES) of Cancer Hallmark gene sets from MSigDB, between samples with high (orange) and low H6PD abundance (green). Size of circle indicates number of proteins in the gene set, and all visualized gene sets adjusted for p value < 0.05. **B** Gene set enrichment analysis showing normalized enrichment score (NES) of Cancer Hallmark gene sets from MSigDB, between samples with high (orange) and low AGR2 abundance (green). Size of circle indicates number of proteins in the gene set, and all visualized gene sets adjusted for p value < 0.05

Both AGR2 and H6PD have been proposed to play a role in breast cancer progression, with elevated levels of these proteins being associated with increased breast cancer cell proliferation and migration [11, 53]. Therefore, we investigated whether high or low H6PD and AGR2 protein expression in breast cancer tissue correlates with the upregulation of pathways that are critical for breast cancer progression (Fig. 4). For this purpose, gene set enrichment analysis was performed to identify enriched pathways in breast cancer tissue based on relatively high and low H6PD and AGR2 protein expression (Fig. 4). Glycolysis, fatty acid metabolism, hypoxia, angiogenesis, and epithelial to mesenchymal transition pathways were enriched in breast cancer tissue exhibiting relatively high H6PD and AGR2 expression. These pathways have been shown to contribute to breast cancer progression, highlighting the importance of the potential AGR2 and H6PD interaction [54–58]. Thus, the interaction of AGR2 and H6PD was assessed in more detail.

### Direct interaction of H6PD and AGR2 in MCF7 cells with endogenous expression of the two proteins

**Fig. 5:**
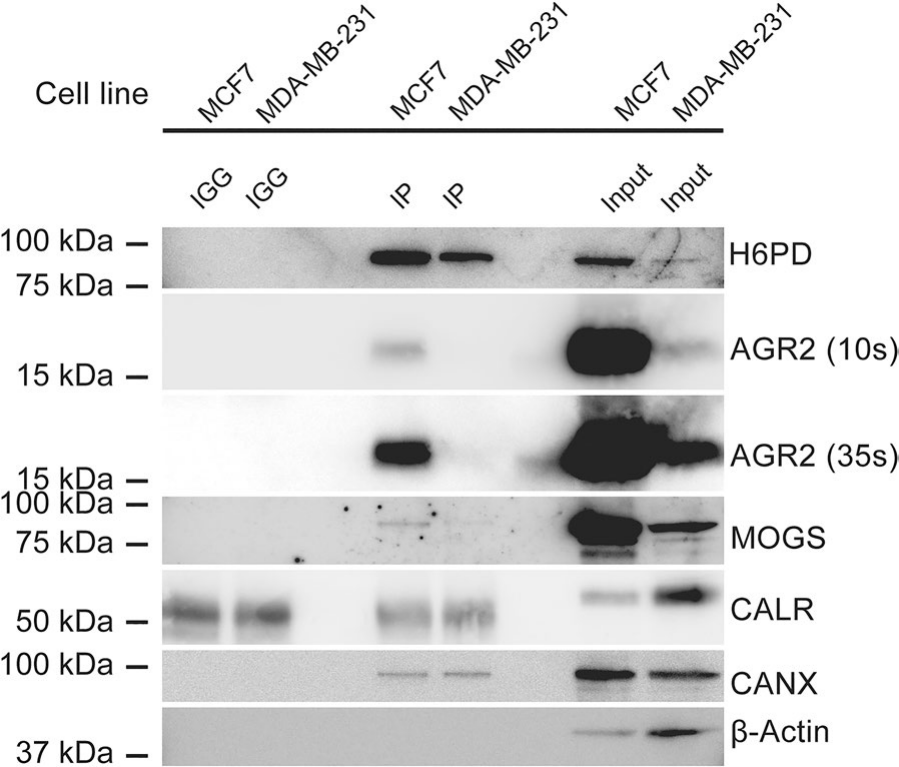
Co-IP of AGR2, MOGS and CANX with H6PD. MCF7 and MDA-MB-231 cells were incubated in the presence of the reversible crosslinking agents DSP and DTME, followed by immunoprecipitation (IP) using anti-H6PD antibody for pull-down and antibodies against AGR2, MOGS, CALR and CANX for the detection of potential interacting partners. β-Actin was used as negative control to exclude nonspecific binding. A representative western blot of three independent experiments is shown

Three different breast cancer cell lines were analyzed for their AGR2 and H6PD protein expression levels by immunoblotting to identify a suitable cell line for Co-IP experiments. The ER^+^,PR^+^,HER2^−^ cell line MCF7 [59] showed high AGR2 protein expression, whereas the TNBC cell lines MDA-MB-231 (H6PD-BirA*-HA clone) and SUM-159 displayed low AGR2 levels that could only be detected after prolonged exposure of the blot (Additional Fig. 1). Therefore, MCF7 cells were chosen for Co-IP experiments, in addition to MDA-MB-231 for which the BioID approach suggested interaction between AGR2 and H6PD. The anti-H6PD antibody was validated for its suitability in IP applications to ensure sufficient antigen binding (Additional Fig. 4). The Co-IP using anti-H6PD antibody revealed a band corresponding to AGR2 following 10 s of exposure in the IP samples derived from MCF7 cells and a very weak band after 35 s of exposure in samples of MDA-MB-231 cells (Fig. 5). No signals for AGR2 protein could be detected in samples incubated with immune globulin G (IGG) control. These results support a physical interaction between H6PD and AGR2.

The glucosidase MOGS [60] and the two lectins CALR and CANX [61, 62] were also found among the 50 most highly enriched biotinylated proteins from MDA-MB-231 cells expressing H6PD-BirA*-HA (Table 1). To test whether H6PD might interact with any of them, the H6PD Co-IP blots were probed with the corresponding antibodies against MOGS, CALR and CANX, respectively (Fig. 5). Probing against MOGS and CANX revealed signals in IP samples from both MCF7 and MDA-MB-231 cells, although the band obtained for MOGS from lysates of MDA-MB-231 cells was weak due to the low expression level, supporting their physical interaction with H6PD. In contrast, no bands at the size of CALR but an unspecific band could be detected that was also present in the IGG control (Fig. 5). β-Actin was probed to exclude non-specific binding of the anti-H6PD antibody used.

Thus, the results from the Co-IP experiments support the findings from the BioID approach suggesting that H6PD physically interacts with AGR2, MOGS and CANX, while this remains unclear for CALR.

### AGR2 regulates H6PD protein expression and enhances its enzyme activity

**Fig. 6:**
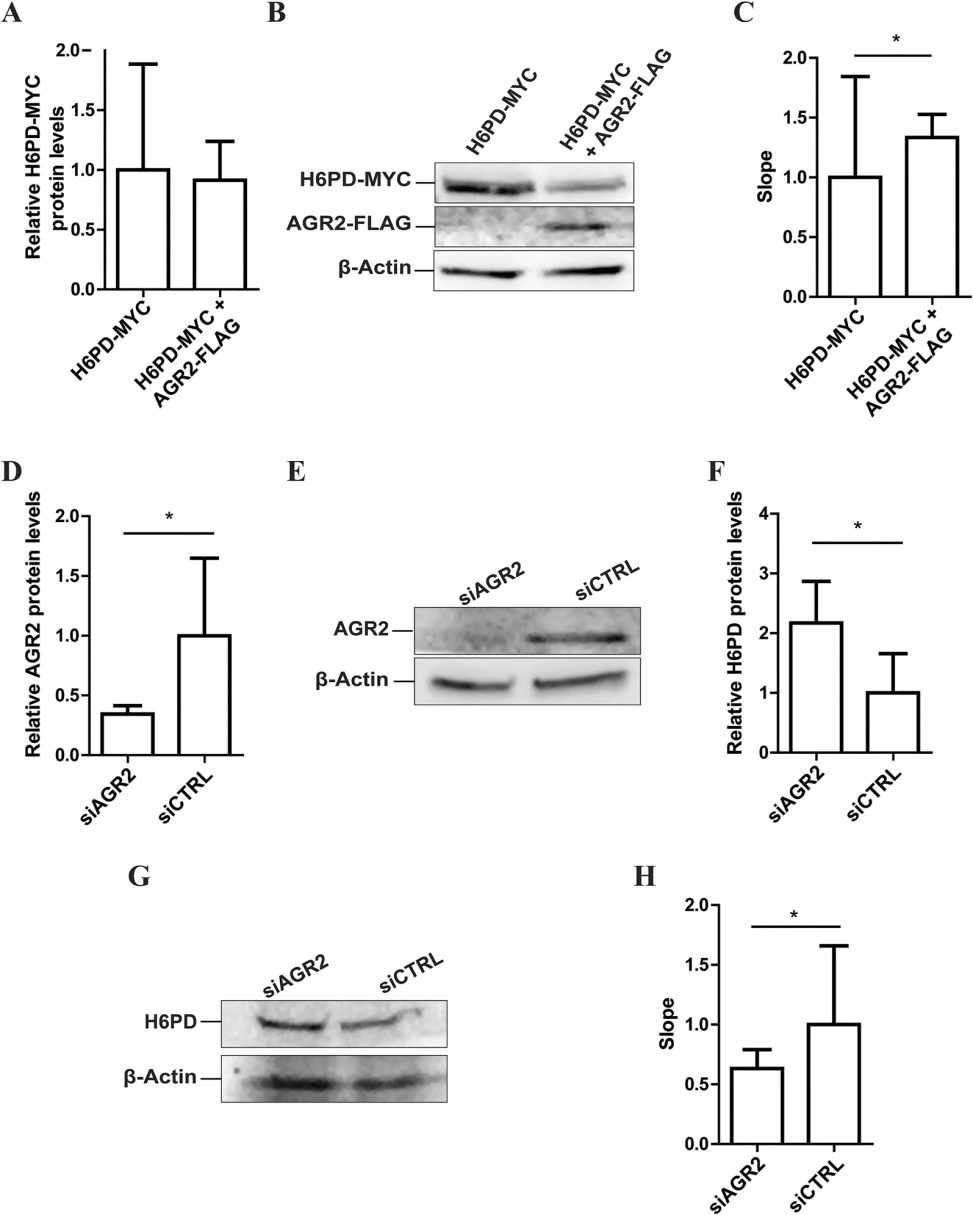
AGR2 regulates H6PD protein expression and enhances its enzyme activity. **A**–**C** HEK-293 cells were transfected with plasmid for H6PD-MYC in the absence or presence of plasmid for AGR2-FLAG, followed by determination of protein expression by immunoblotting and measurement of H6PD enzyme activity. A) Relative H6PD-MYC protein levels in microsomes of HEK-293 cells normalized to the β-Actin loading control, determined by densitometry analysis. H6PD-MYC expression was to β-Actin protein levels, relative to that of microsomes of H6PD-MYC transfected cells. **B** Representative western blot analysis of H6PD-MYC and AGR2-FLAG protein levels in microsomes of HEK-293 cells transfected with H6PD-MYC alone or together with AGR2-FLAG. Membranes were probed with anti-MYC antibody for H6PD and anti-FLAG antibody for AGR2 detection. **C** H6PD activity in microsomes of HEK-293 cells transfected with H6PD-MYC alone or together with AGR2-FLAG. In each independent experiment, the results were normalized to the activity of microsomes of HEK-293 cells transfected with H6PD-MYC. D-H) MCF7 cells were treated for 72 h with control siRNA (siCTRL), siRNA against AGR2 (siAGR2) or H6PD (siH6PD), followed by immunoblotting analysis and determination of H6PD activity in microsomal preparations. **D** AGR2 protein levels were normalized to the β-Actin loading control and are relative to siCTRL, determined by densitometry analysis. E) Representative western blot of (**D**). **F** H6PD protein levels normalized to β-Actin loading control and relative to siCTRL. **G** Representative western blot of (**F**). **H** H6PD activity in microsomes of MCF7 cells transfected with siCTRL or siAGR2. In each independent experiment, results were normalized to H6PD activity in microsomes of siCTRL-transfected MCF7 cells. **A**, **C**, **D**, **F**, **H** Data represent mean ± SD from three independent experiments. Mann–Whitney U test was performed, p-value: * = < 0.05

Next, a possible direct effect of AGR2 on H6PD protein expression was assessed by transfecting HEK-293 cells with a plasmid encoding H6PD-MYC in the absence or presence of a plasmid encoding AGR2-FLAG. Microsomes were isolated and H6PD-MYC expression was analyzed by immunoblotting, showing no substantial effect following co-expression with AGR2 (Fig. 6A, B, Additional Fig. 5 A–C). Expression of AGR2-FLAG protein was verified by western blot analysis (Fig. 6B). H6PD activity was then measured using G6S as substrate, as described in the methods section, and by recording the absorbance at 340 nm, corresponding to the formation of NADPH. Data were normalized relative to H6PD-MYC expression in the respective microsomal fraction. Linear regression lines were normalized to the X = 0 data point in each analyzed group, representing the baseline of H6PD-dependent NADPH formation. H6PD activity was then compared between cells expressing H6PD-MYC and cells co-expressing H6PD-MYC and AGR2-FLAG by examining the slopes of the linear regression lines derived from the data points obtained (Additional Fig. 5D–F). The results revealed a significantly steeper slope for cells co-expressing AGR2-FLAG and H6PD-MYC (Fig. 6C), suggesting that AGR2 promotes H6PD activity by enhancing the fraction of enzymatically active H6PD molecules.

To support above observations, we tested whether AGR2 knockdown would affect H6PD activity in a cell line with endogenous AGR2 and H6PD expression. For this purpose, MCF7 cells endogenously expressing both proteins (Additional Fig. 1) were transfected with siRNA targeted against AGR2 (siAGR2) or scrambled control siRNA (siCTRL). Successful AGR2 knockdown was verified by immunoblotting and densitometry analysis (Fig. 6D, E Additional Figs. 6 and 7 A–C). Significantly elevated H6PD protein expression was observed 72 h post-transfection of MCF7 cells with siAGR2 (Additional Fig. 6). In contrast, H6PD knockdown did not affect AGR2 expression levels. To assess H6PD enzyme activity, microsomes were isolated 72 h post-transfection and NADPH generation after adding G6S substrate was determined by measuring absorbance at 340 nm using fresh samples. The obtained activities were normalized to H6PD expression (Fig. 6F, G, Additional Fig. 7 A–C). The linear regression curves were normalized to X = 0 as above, revealing significantly lower H6PD activity following AGR2 knockdown (Fig. 6H, Additional Fig. 7D–F).

Thus, we identified a novel H6PD interactor, *i.e.* AGR2, started to explore breast cancer hallmark pathways to which this interaction might contribute to, and provided evidence that AGR2 enhances H6PD activity.

## Discussion

A decade ago, Roux and colleagues developed BioID, a tool for proximity labeling of proteins in living mammalian cells [63]. Several studies then affirmed the applicability of BioID for the elucidation of protein interactomes. BioID was successfully deployed not only to study mammalian cells but also for examinations of protein interactomes in plants, viruses, bacteria, protozoa and even in living animals [64–69]. Here, we applied the BioID approach within the endoplasmic reticulum, a challenging compartment to investigate, as it cannot be isolated in an intact manner and due to its more oxidative environment compared to the cytosol. To verify that the BioID method works in this compartment, we first confirmed using a MDA-MB-231 clone co-expressing H6PD-BirA*-HA and 11β-HSD1-FLAG the biotinylation of 11β-HSD1, the so far only demonstrated direct interactor of the luminal enzyme H6PD [21, 22], before searching for novel interacting partners of this enzyme.

We used the TNBC line MDA-MB-231 as a model system for the generation of a cell clone stably expressing H6PD-BirA*-HA because of our interest to start elucidating the role of H6PD and the luminal PPP in breast cancer cells and due to the absence of endogenous 11β-HSD1 expression in these cells. Thorough validation of luminal biotinylation was followed by MS analysis of biotinylated proteins, and the specificity of the approach was underpinned by the fact that 45 of the 50 most highly enriched proteins were found to be spatially associated with the endoplasmic reticulum. The remaining five included a secretory protein, two plasma membrane proteins and two lysosomal proteins, thus proteins that may pass the endoplasmic reticulum during their maturation.

Due to the reported role of AGR2 in breast cancer [70, 71] and our observations of the impact of H6PD on breast cancer cell properties [11], we selected AGR2 from the pool of potential interactors for further investigations. An earlier study exploring the interactome of AGR2 by using Co-IP [72] found several interacting partners that were identified also in the present study as biotinylated hits and potential interactors of H6PD, *i.e.* PDIA3, CANX and CALR, suggesting involvement of H6PD and AGR2 in the CALR/CANX cycle. The CALR/CANX cycle has important effects on Ca^2+^-homeostasis and functional crosstalk between mitochondria and endoplasmic reticulum [73], where H6PD and AGR2 might play a role in modulating fatty acid metabolism and glycolysis that requires further investigations. Other shared potential interactors include PDIA6, ERO1 A, ERP29, PRXV and HSPA5, proposing a role of H6PD and AGR2 in UPR-mediated signaling, chaperone activation and protein folding. This suggests that H6PD and AGR2 are part of a multi-protein complex involved in protein folding control, where H6PD might provide the redox equivalent NADPH. However, the proteins utilizing NADPH for this purpose remain to be identified.

We had anticipated identifying enzymes bearing a nucleotide-binding site and using NADPH among the biotinylated proteins. However, none of the top 50 enriched biotinylated proteins contains a Rossmann-fold motif or is known to bind NAD(P)(H). The absence of NADPH-binding luminal interacting partners such as other dehydrogenases/reductases suggests that a direct physical interaction, as seen with 11β-HSD1 [21], is not required for their function.

The BioID method has the advantage of detecting also weak and transient protein–protein interactions as well as proteins in the close neighborhood of the bait protein that may not necessarily directly interact with the bait. Thus, confirmation using a second, independent method is important. The interaction between H6PD and AGR2 was confirmed by Co-IP from MCF7 cells coexpressing endogenous levels of both proteins. The same was true for MOGS and CANX but not CALR, where CO-IP failed to detect an interaction. Because CALR is known to interact with CANX [73], a likely explanation for its biotinylation by H6PD-BirA*-HA is indirectly in a protein complex but in close proximity to the biotin ligase fused to H6PD. To prove a direct protein–protein interaction between H6PD and AGR2, purified proteins and methods like surface plasmon resonance using appropriate controls to exclude unspecific interactions will need to be applied in future studies.

Besides providing evidence that H6PD and AGR2 can contribute to a multi-protein complex, our results propose functional interactions between the two proteins. First, co-expression of AGR2 with H6PD in HEK-293 cells enhanced H6PD activity, without substantially altering its expression levels. Second, the siRNA-mediated reduction in AGR2 expression decreased H6PD activity but led to increased H6PD protein expression in MCF7 cells, suggesting that AGR2 plays a role in the regulation of the fraction of enzymatically active H6PD molecules.

This seems unlikely to be caused by altered protein half-life. Our preliminary experiments suggest a protein half-life for AGR2 of < 2 h, in line with earlier observations [74, 75], independent of the absence or presence of H6PD when measured in HEK-293 cells. H6PD showed a protein half-life > 36 h, independent whether AGR2 was coexpressed or not. However, because the half-life of the two proteins differ a lot, a different experimental approach should be applied, using inducible knockout of either one of the genes in a cell line with endogenous expression.

Another mechanism how AGR2 could enhance H6PD activity includes its function as a PDI. AGR2 functions as a dimer and, interestingly, seven proteins identified in our screening for H6PD interactors were reported as candidates regulating AGR2 dimerization, *i.e.* HYOU1, ERO1 A and HSP90B1, representing candidate AGR2 homodimer inhibitors, and TMED2, LMAN1, UGGT1 and H6PD, proposed to function as AGR2 homodimer enhancers [76]. AGR2 plays a role in folding cysteine-rich proteins by forming mixed disulfides with protein substrates and subsequently re-arranging disulfide bonds [77, 78]. The amino acid sequence of H6PD (Sequence: NM_001282587.2) contains 10 cysteine residues, which could potentially form an interaction with AGR2 via mixed disulfides. Whether a mixed disulfide with AGR2 is involved in the observed increase in H6PD activity or whether H6PD affects AGR2 activity requires further research.

The gene set enrichment analysis provided initial insight into pathways where the AGR2 stimulated H6PD activity might be relevant. These included, among others, pathways related to energy adaptation in breast cancer, such as fatty acid metabolism and glycolysis that were found to be enriched in breast cancer tissues, with elevated AGR2 or H6PD expression [54, 55]. An elevated H6PD-dependent NADPH generation in the endoplasmic reticulum lumen may enhance 17β-HSD7 activity, which has its catalytic site facing this compartment [48], to promote cholesterol synthesis and tumor growth [79, 80]. Another enzyme using NADPH in the endoplasmic reticulum lumen is 17β-HSD12, with a role in very long-chain fatty acid elongation [81]. High 17β-HSD12 expression correlated with poor prognosis for survival in breast cancer and ovarian cancer patients [82–84]. However, an effect of H6PD on the activities of these enzymes has not yet been demonstrated due to the lack of suitable cell-based bioassays in absence or presence of H6PD.

Breast cancer cells adjust to the high energy demand by inducing metabolic adaptation, which is considered a hallmark of breast cancer progression. It is known that these cells increase their rate of glycolysis to meet the increased energy demand associated with proliferation and migration [54, 85, 86]. Previous research indicated that knockdown of AGR2 in human RL952 endometrial carcinoma cells resulted in the downregulation of genes related to glycolysis and a decrease in lactate production, indicating an impairment of glycolysis [87]. Moreover, knockdown of AGR2 in NT2-D1 patient-derived testicular carcinoma cells decreased lactate levels, whilst AGR2 overexpression increased lactate production, confirming a role of AGR2 in glycolysis [88].

H6PD is directly linked to glucose metabolism via the luminal PPP. Our earlier results showed that knockdown of H6PD in breast cancer cell lines reduces proliferation and/or migration [11]. This suggests that H6PD activity and thus luminal PPP promotes an aggressive cancer cell phenotype in different subtypes. Our preliminary data indicate that H6PD knockout in MCF7 cells reduces lactate production. These observations suggest that AGR2 and H6PD may work in a complex to enhance glycolytic activity. Whether inhibition of H6PD might substantially suppress glycolysis in breast tumors and inhibit cancer progression remains unclear, and a detailed analysis of the impact of H6PD on energy metabolism as well as on redox regulation is required for a better understanding of the role of this enzyme in modulating cancer progression.

In conclusion, BioID is a useful tool for the screening of interacting proteins in the endoplasmic reticulum lumen and represents a valuable method to expand our understanding of this yet insufficiently understood cellular compartment. Nevertheless, validation of potential interactors using a second method such as Co-IP or immune-fluorescence based approaches is crucial because false positive hits are of concern when using BioID to identify interacting proteins [89, 90]. This is especially important to exclude endogenously biotinylated proteins formed by biotin-dependent carboxylases [91]. The present study revealed AGR2 as an interacting partner of H6PD that enhances its enzymatic activity. Follow-on experiments are warranted to uncover the exact mechanism how AGR2 stimulates H6PD activity, thereby contributing to a more aggressive cancer cell phenotype. Gene set enrichment analysis highlighted enhanced H6PD and AGR2 expression in pathways such as fatty acid metabolism and glycolysis that have key roles in cancer progression and deserve special attention in further research.

## Supplementary Information

Supplementary Material 1.

Supplementary Material 2.

Supplementary Material 3.

Supplementary Material 4.

Supplementary Material 5.

Supplementary Material 6.

Supplementary Material 7.

Supplementary Material 8.

Supplementary Material 9.

## Data Availability

The datasets supporting the conclusions of this article are included in the article and its additional files. The data generated during and/or analyzed during the current study are available at Zenodo: https://doi.org/10.5281/zenodo.14653475 (Sakalauskaite, G., Weingartner, M., Ebert, S., Boot, G., Bock, T., Birk, J., Tsachaki, M., Gallon, J., Piscuoglio, S., & Odermatt, A. (2025). Data-Sets_A BioID-based approach uncovers the interactome of hexose-6-phosphate dehydrogenase in breast cancer cells and identifies anterior gradient protein 2 as an interacting partner [Data set]). All mass spectrometry raw data files associated with this manuscript are accessible at MassIVE (https://massive.ucsd.edu) under accession number MSV000096699.
