# Supplementary figures and images for: A BioID-based approach uncovers the interactome of hexose-6-phosphate dehydrogenase in breast cancer cells and identifies anterior gradient protein 2 as an interacting partner
Source: Cell Biosci. 2025 Apr 25;15:54. doi: 10.1186/s13578-025-01388-9 (PMC12032772; doi:10.1186/s13578-025-01388-9)

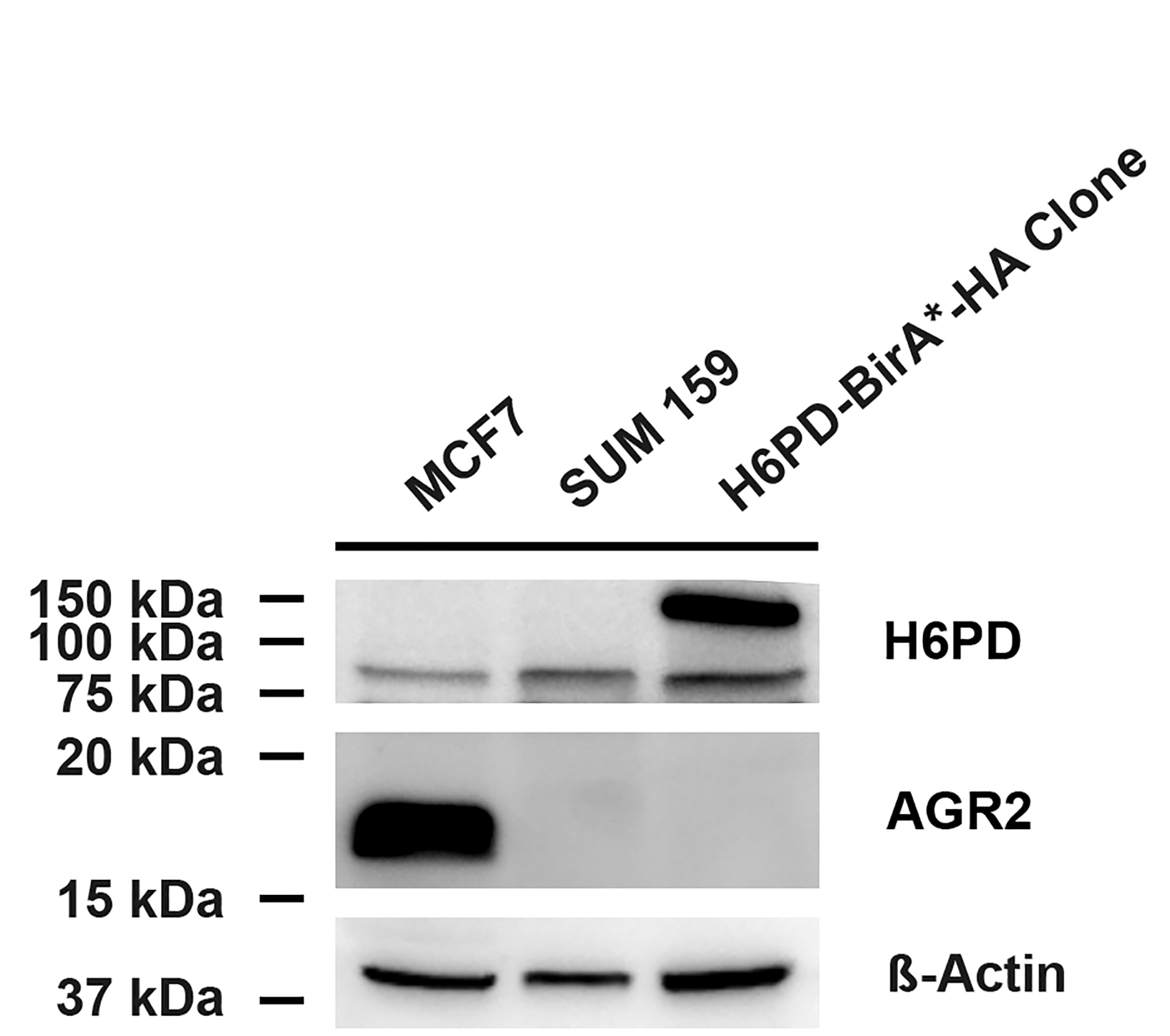

Supplement: Supplementary file 1 — Supplementary Material 1. [file 13578_2025_1388_MOESM1_ESM.tif]

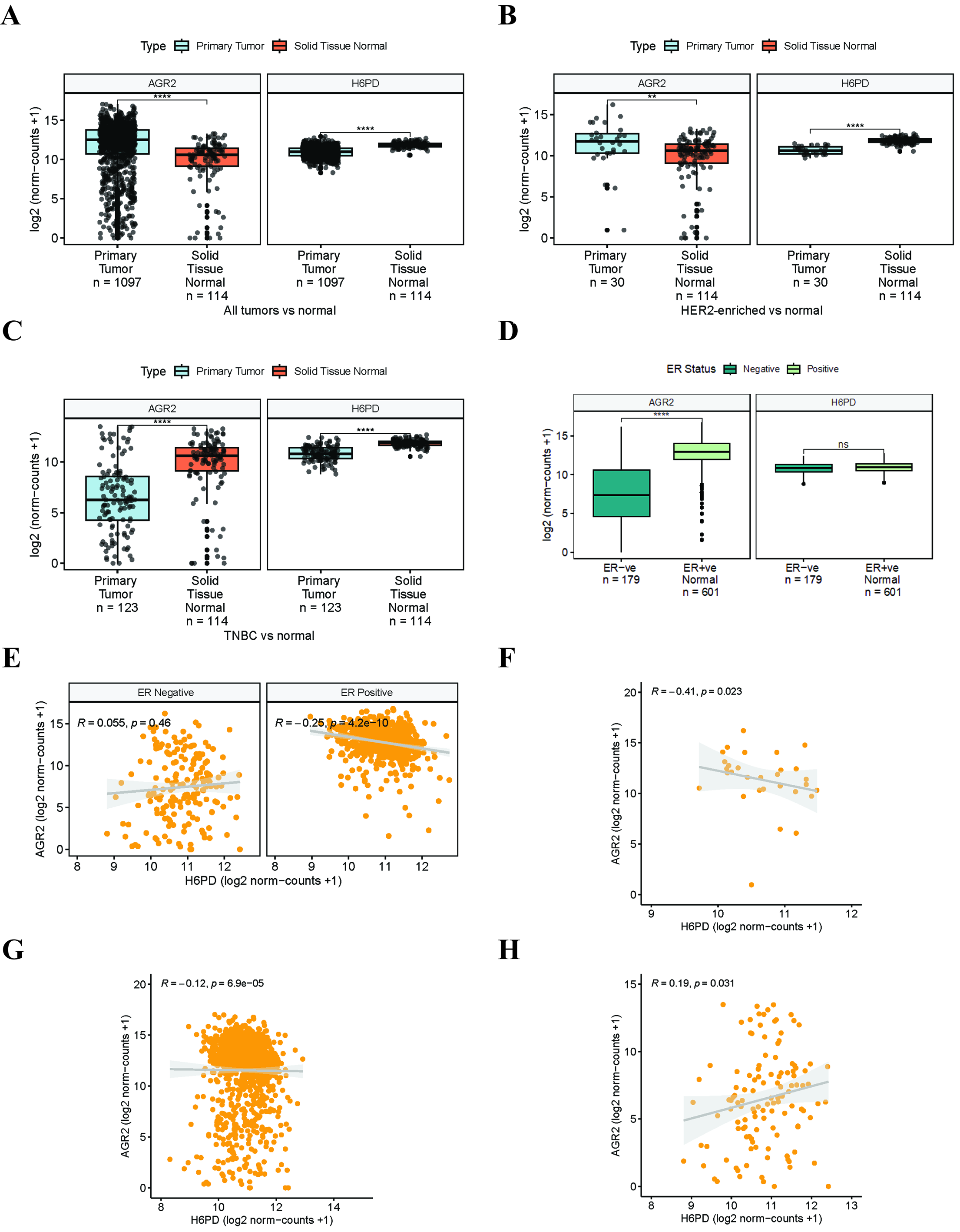

Supplement: Supplementary file 2 — Supplementary Material 2. [file 13578_2025_1388_MOESM2_ESM.tif]

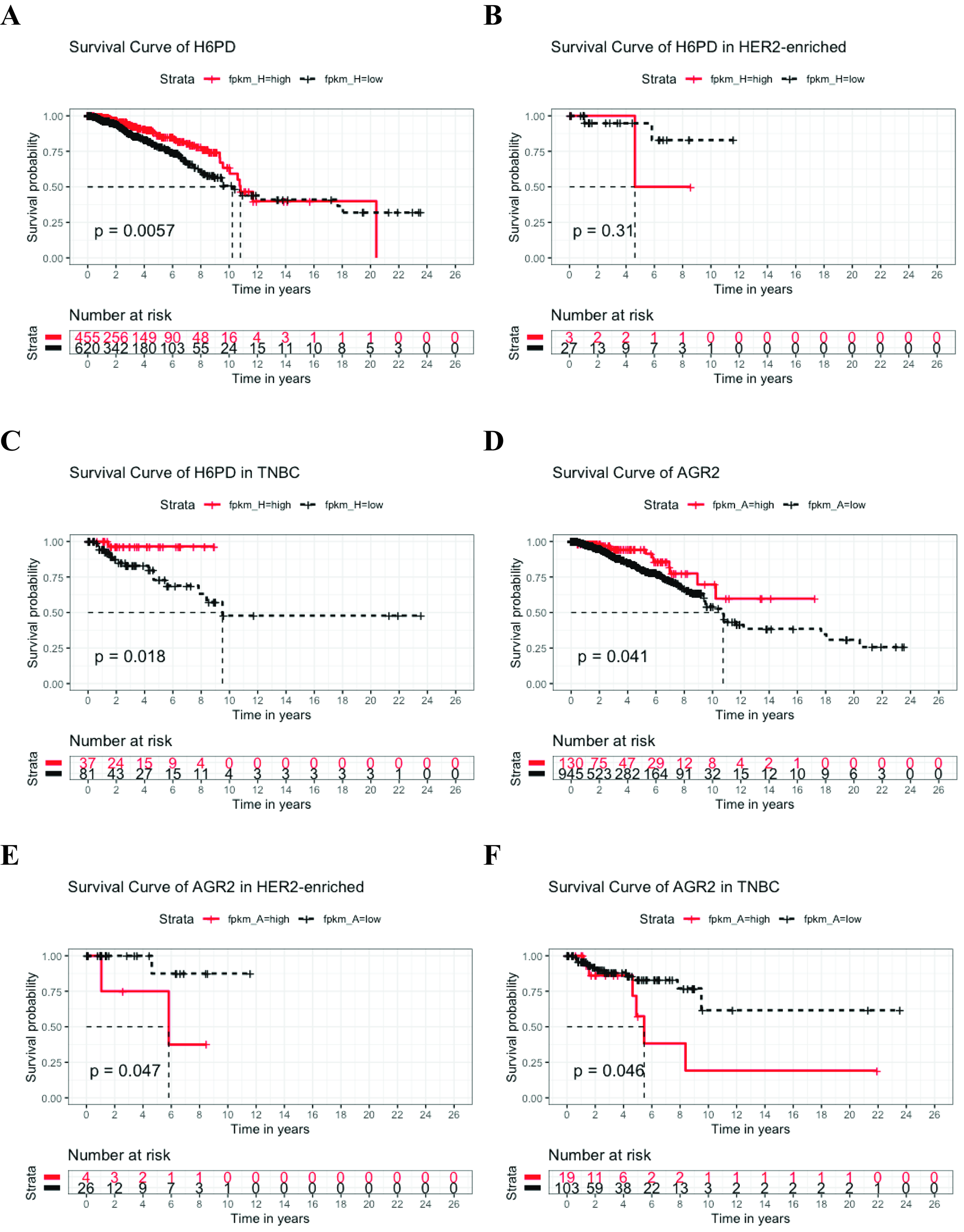

Supplement: Supplementary file 3 — Supplementary Material 3. [file 13578_2025_1388_MOESM3_ESM.tif]

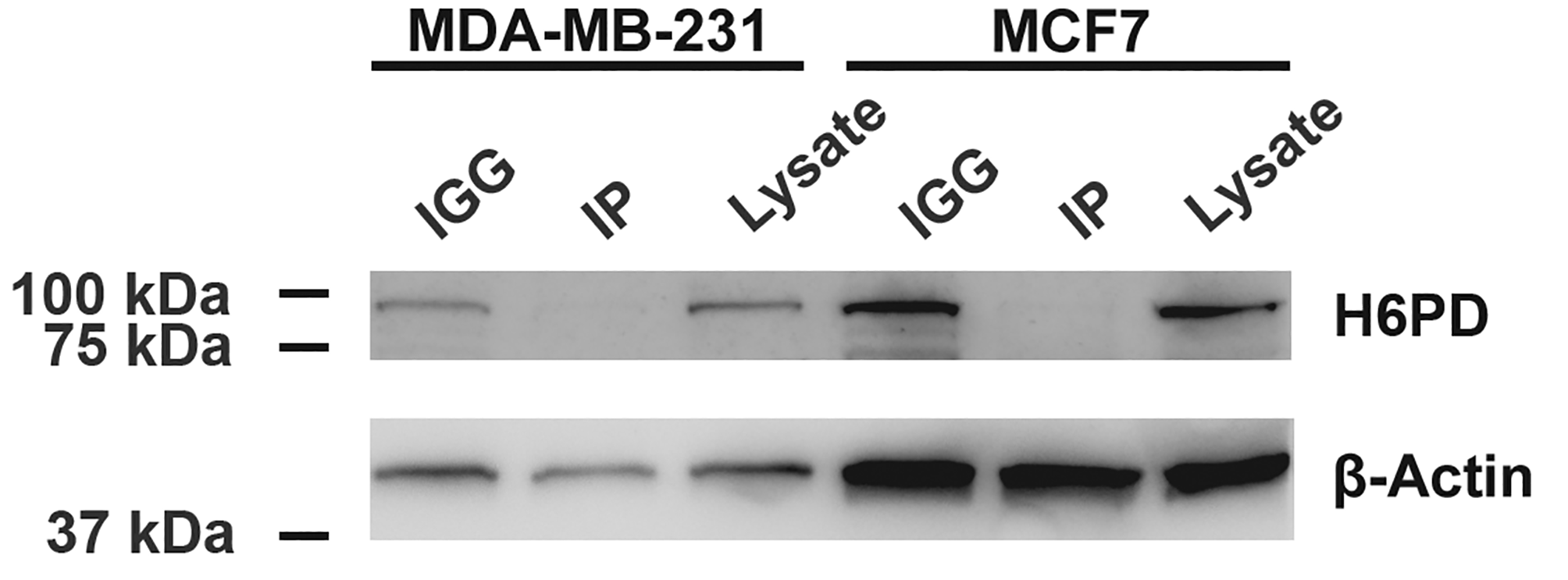

Supplement: Supplementary file 4 — Supplementary Material 4. [file 13578_2025_1388_MOESM4_ESM.tif]

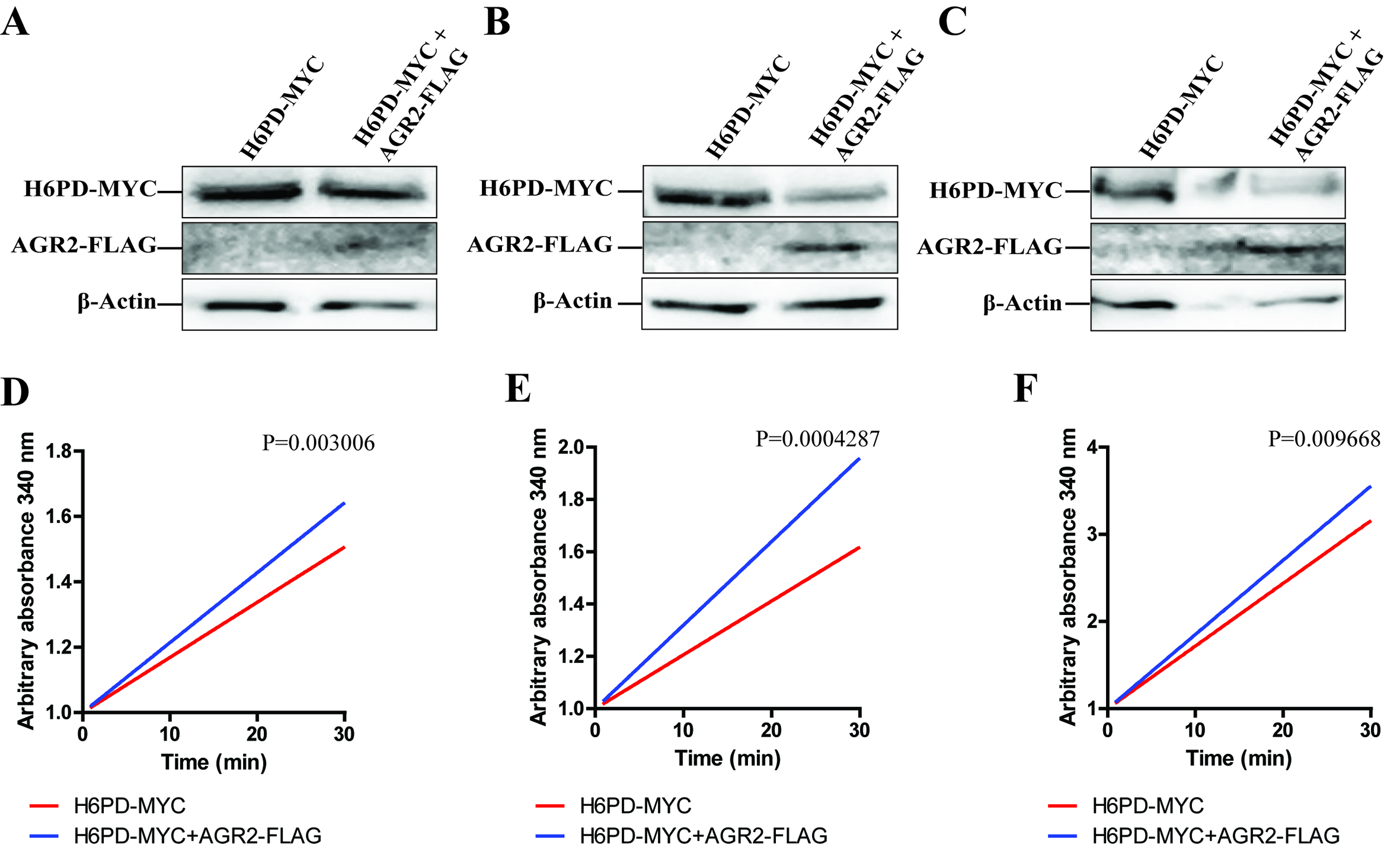

Supplement: Supplementary file 5 — Supplementary Material 5. [file 13578_2025_1388_MOESM5_ESM.tif]

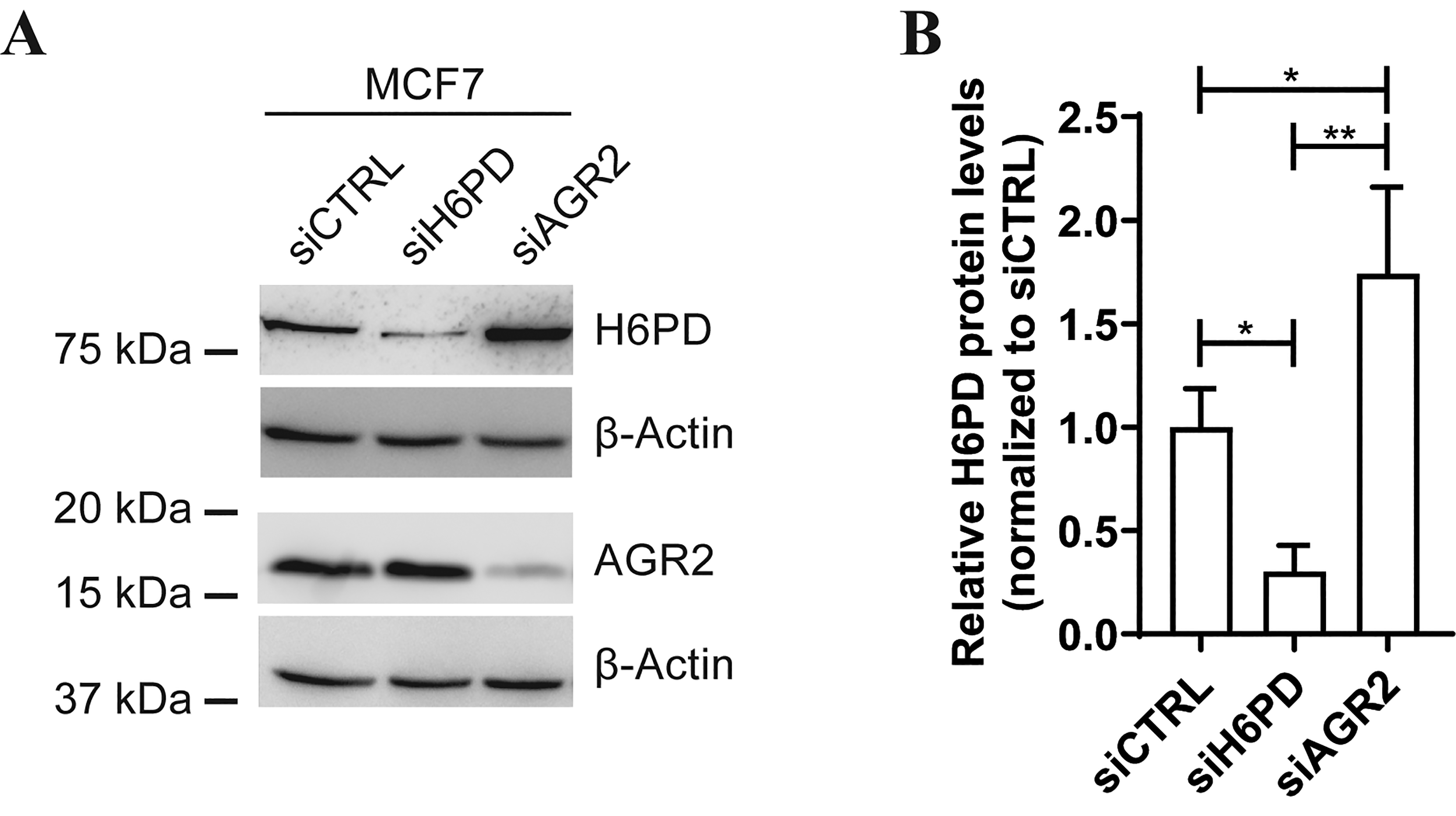

Supplement: Supplementary file 6 — Supplementary Material 6. [file 13578_2025_1388_MOESM6_ESM.tif]

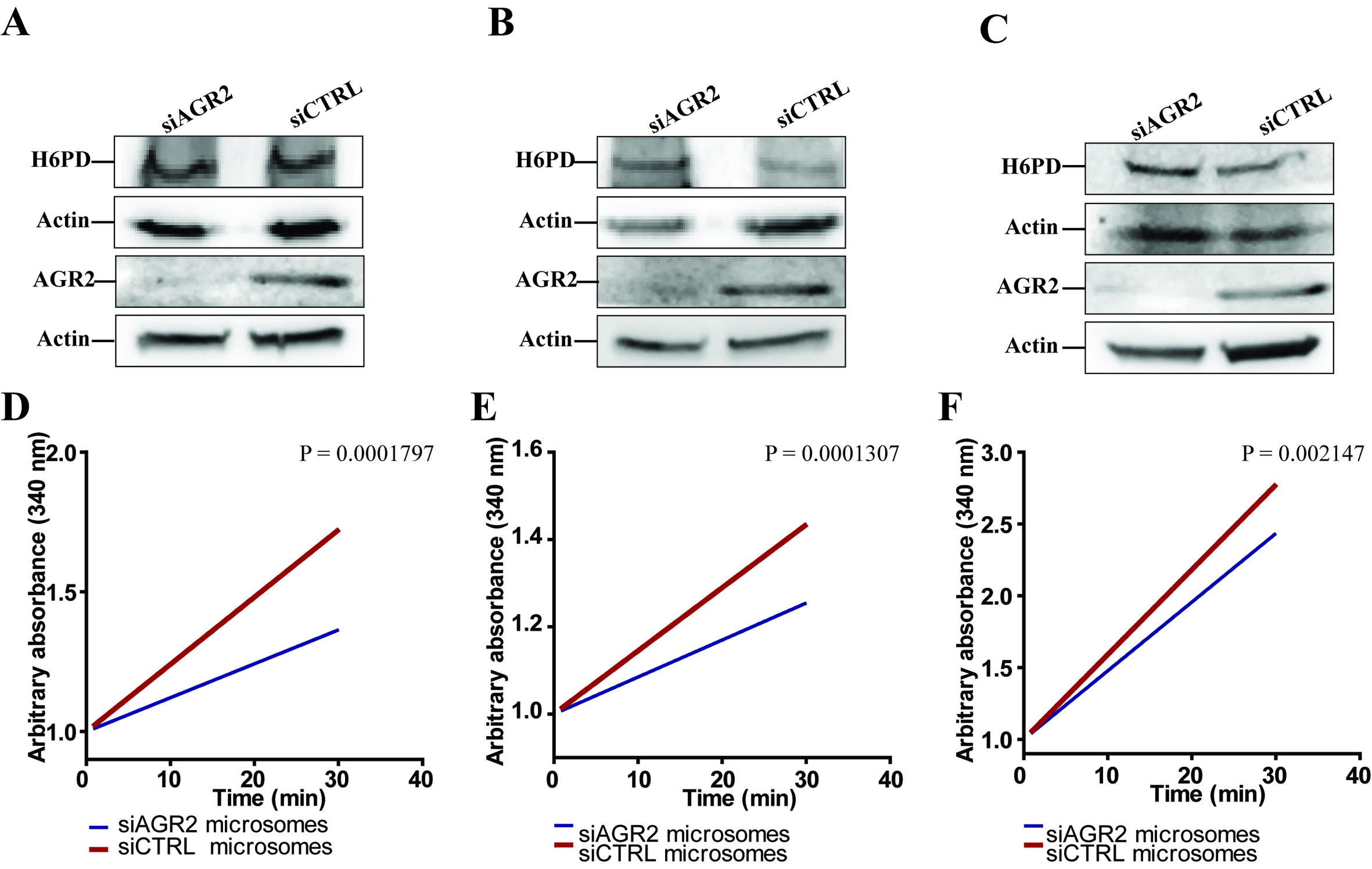

Supplement: Supplementary file 7 — Supplementary Material 7. [file 13578_2025_1388_MOESM7_ESM.tif]
