# Supplementary material for: A BioID-based approach uncovers the interactome of hexose-6-phosphate dehydrogenase in breast cancer cells and identifies anterior gradient protein 2 as an interacting partner
Source: Cell Biosci. 2025 Apr 25;15:54. doi: 10.1186/s13578-025-01388-9 (PMC12032772; doi:10.1186/s13578-025-01388-9)
Supplement: Supplementary file 9 — Supplementary Material 9. [file 13578_2025_1388_MOESM9_ESM.docx]

**Additional Information**

**Additional Tables**

**Additional Table 1**

**Sequence of the H6PD-BirA*-HA construct.**

The sequences of the different motifs are shown as follows: Hexose-6-phosphate dehydrogenase (H6PD) – Linker (blue) - promiscuous biotin ligase (BirA*) (magenta) - human influenza hemagglutinin epitope (HA) (orange).

| **Sequence of H6PD-BirA*-HA** |
| --- |
| MWNMLIVAMCLALLGCLQAQELQGHVSIILLGATGDLAKKYLWQGLFQLYLDEAGRGHSFSFHGAALTAPKQGQELMAKALESLSCPKDMAPSHCAEHKDQFLQLSQYRQLKTAEDYQALNKDIEAQLQHAGLREAGRIFYFSVPPFAYEDIARNINSSCRPGPGAWLRVVLEKPFGHDHFSAQQLATELGTFFQEEEMYRVDHYLGKQAVAQILPFRDQNRKALDGLWNRHHVERVEIIMKETVDAEGRTSFYEEYGVIRDVLQNHLTEVLTLVAMELPHNVSSAEAVLRHKLQVFQALRGLQRGSAVVGQYQSYSEQVRRELQKPDSFHSLTPTFAAVLVHIDNLRWEGVPFILMSGKALDERVGYARILFKNQACCVQSEKHWAAAQSQCLPRQLVFHIGHGDLGSPAVLVSRNLFRPSLPSSWKEMEGPPGLRLFGSPLSDYYAYSPVRERDAHSVLLSHIFHGRKNFFITTENLLASWNFWTPLLESLAHKAPRLYPGGAENGRLLDFEFSSGRLFFSQQQPEQLVPGPGPAPMPSDFQVLRAKYRESPLVSAWSEELISKLANDIEATAVRAVRRFGQFHLALSGGSSPVALFQQLATAHYGFPWAHTHLWLVDERCVPLSDPESNFQGLQAHLLQHVRIPYYNIHPMPVHLQQRLCAEEDQGAQIYAREISALVANSSFDLVLLGMGADGHTASLFPQSPTGLDGEQLVVLTTSPSQPHRRMSLSLPLINRAKKVAVLVMGRMKREITTLVSRVGHEPKKWPISGVLPHSGQLVWYMDYDAFLGGSKDNTVPLKLIALLANGEFHSGEQLGETLGMSRAAINKHIQTLRDWGVDVFTVPGKGYSLPEPIQLLNAKQILGQLDGGSVAVLPVIDSTNQYLLDRIGELKSGDACIAEYQQAGRGGRGRKWFSPFGANLYLSMFWRLEQGPAAAIGLSLVIGIVMAEVLRKLGADKVRVKWPNDLYLQDRKLAGILVELTGKTGDAAQIVIGAGINMAMRRVEESVVNQGWITLQEAGINLDRNTLAAMLIRELRAALELFEQEGLAPYLSRWEKLDNFINRPVKLIIGDKEIFGISRGIDKQGALLLEQDGIIKPWMGGEISLRSAEKAYPYDVPDYA |

**Additional Table 2**

**Primary and secondary antibodies used in this study.**

List of the targets of primary and secondary antibodies, along with their catalog number and supplier.

| **Primary Antibody** | Catalog number | Company |
| --- | --- | --- |
| Polyclonal rabbit anti-H6PD antibody | HPA004824 | Sigma-Aldrich |
| monoclonal mouse anti-H6PD antibody | sc-377180 | Santa Cruz Biotechnology |
| monoclonal mouse anti-AGR2 antibody | sc-101211 | Santa Cruz |
| Streptavidin-HRP | 21130 | Thermo Fisher Scientific |
| monoclonal mouse anti-β-Actin antibody | sc-47778 | Santa Cruz Biotechnology |
| monoclonal rabbit anti-Lamin B1 antibody | ab133741 | Abcam |
| rabbit IgG Isotope control | 10500C | Thermo Fisher Scientific |
| monoclonal mouse anti-MOGS | sc-374006 | Santa Cruz Biotechnology |
| polyclonal rabbit anti-Calnexin antibody | SAB4503258 | Sigma Aldrich |
| polyclonal rabbit anti-Calreticulin antibody | 2891 | Cell Signaling Technology |
| monoclonal mouse anti-FLAG antibody | F1804 | Sigma Aldrich |
| monoclonal rat anti-HA antibody | 11867423001 | Roche |
| monoclonal mouse anti-MYC Antibody | 05-724 | Sigma Aldrich |

| **Secondary antibody** | Catalog number | Company |
| --- | --- | --- |
| HRP-conjugated goat anti-mouse antibody | A0168 | Sigma Aldrich |
| HRP-conjugated goat anti-rabbit antibody | A0545 | Sigma Aldrich |
| HRP-conjugated goat anti-rat antibody | 7077S | Cell signaling |
| Alexa Fluor™ 555 Goat anti-Rat | A-21434 | Invitrogen |
| Alexa Fluor™ 488 Goat anti-Rabbit | A11008 | Invitrogen |

**Additional Table 3 (Additional_Table3.xlsx)**

**Biotinylated proteins identified by mass spectrometry after immunoprecipitation using streptavidin-labeled magnetic beads.**

The presented log_2_-fold change represents enrichment of identified peptides in H6PD-BirA*-HA clone compared to MDA-MB 231 control samples. The numbers of peptides used for quantification of each protein are listed in the “Peptides” column. Gene function and subcellular localization were adapted from UniProt (24^th^ December 2020) and sources cited in this work. The data presented were obtained from three independent experiments (see separately uploaded file **Additional_Table3.xlsx**).

**Additional Table 4**

**STRING database pathway analysis of the 50 most significant proteins detected in MDA-MB-231 cells.**

Reactome pathway number, pathway description, number of pathway-matching proteins identified with BioID approach, strength and false discovery rate of the analysis are provided in the table. Pathways particularly interesting in context of contribution to breast cancer progression are highlighted in green.

| **Pathway number** | **Description** | **Count in network** | **Strength** | **False discovery rate** |
| --- | --- | --- | --- | --- |
| HSA-168316 | Assembly of Viral Components at the Budding Site | 2 of 2 | 2.6 | 0.0045 |
| HSA-9683686 | Maturation of spike protein | 3 of 5 | 2.37 | 0.00018 |
| HSA-3000484 | Scavenging by Class F Receptors | 2 of 6 | 2.12 | 0.0153 |
| HSA-381183 | ATF6 (ATF6-alpha) activates chaperone genes | 3 of 10 | 2.07 | 0.00061 |
| HSA-532668 | N-glycan trimming in the ER and Calnexin/Calreticulin cycle | 7 of 35 | 1.9 | 2.48E-08 |
| HSA-901042 | Calnexin/Calreticulin cycle | 5 of 26 | 1.88 | 5.21E-06 |
| HSA-1679131 | Trafficking and processing of endosomal TLR | 2 of 13 | 1.78 | 0.0461 |
| HSA-983170 | Antigen Presentation: Folding, assembly and peptide loading of class I MHC | 4 of 29 | 1.74 | 0.00027 |
| HSA-5694530 | Cargo concentration in the ER | 4 of 33 | 1.68 | 0.0004 |
| HSA-381070 | IRE1alpha activates chaperones | 6 of 50 | 1.67 | 2.60E-06 |
| HSA-381038 | XBP1(S) activates chaperone genes | 5 of 48 | 1.61 | 5.06E-05 |
| HSA-381119 | Unfolded Protein Response (UPR) | 8 of 92 | 1.53 | 8.36E-08 |
| HSA-9694548 | Maturation of spike protein | 3 of 37 | 1.5 | 0.0129 |
| HSA-2173782 | Binding and Uptake of Ligands by Scavenger Receptors | 3 of 43 | 1.44 | 0.0182 |
| HSA-8957275 | Post-translational protein phosphorylation | 7 of 107 | 1.41 | 5.21E-06 |
| HSA-204005 | COPII-mediated vesicle transport | 4 of 68 | 1.37 | 0.0042 |
| HSA-381426 | Regulation of Insulin-like Growth Factor (IGF) transport and uptake by Insulin-like Growth Factor Binding Proteins (IGFBPs) | 7 of 124 | 1.35 | 9.61E-06 |
| HSA-1650814 | Collagen biosynthesis and modifying enzymes | 3 of 67 | 1.25 | 0.0498 |
| HSA-446203 | Asparagine N-linked glycosylation | 12 of 304 | 1.19 | 2.48E-08 |
| HSA-199977 | ER to Golgi Anterograde Transport | 5 of 154 | 1.11 | 0.0052 |
| HSA-114608 | Platelet degranulation | 4 of 126 | 1.1 | 0.0257 |
| HSA-168255 | Influenza Infection | 4 of 155 | 1.01 | 0.0483 |
| HSA-2262752 | Cellular responses to stress | 11 of 747 | 0.76 | 0.0004 |
| HSA-597592 | Post-translational protein modification | 20 of 1405 | 0.75 | 5.66E-08 |
| HSA-5653656 | Vesicle-mediated transport | 9 of 666 | 0.73 | 0.0047 |
| HSA-168256 | Immune System | 14 of 1979 | 0.45 | 0.025 |
